# Multi-timescale neural adaptation underlying long-term musculoskeletal reorganization

**DOI:** 10.7554/eLife.108684

**Published:** 2026-06-19

**Authors:** Roland Philipp, Yuki Hara, Naohito Ohta, Naoki Uchida, Tomomichi Oya, Tetsuro Funato, Kazuhiko Seki

**Affiliations:** 1 National Center of Neurology and Psychiatry, Department of Neurophysiology Tokyo Japan; 2 University of ElectroCommunications, Graduate School of Informatics and Engineering, Department of Mechanical and Intelligent Systems Engineering Tokyo Japan; 3 National Center of Neurology and Psychiatry, Department of Orthopaedic Surgery Tokyo Japan; 4 Western Institute for Neuroscience, University of Western Ontario London Canada; Champalimaud Foundation Portugal; University of Cambridge United Kingdom

**Keywords:** tendon transfer, muscle synergy, neural adaptation, tenodesis, motor control, *Macaca fuscata*, Other

## Abstract

The central nervous system (CNS) can effectively control body movements despite environmental changes. While much is known about adaptation to external environmental changes, less is known about responses to internal bodily changes. This study investigates how the CNS adapts to long-term alterations in the musculoskeletal system using a tendon transfer model in nonhuman primates (*Macaca fuscata*). We surgically relocated finger flexor and extensor muscles to examine how the CNS adapts its strategy for finger movement control by measuring muscle activities during grasping tasks. Two months post-surgery, the monkeys demonstrated significant recovery of grasping function despite the initial disruption. Our findings suggest a two-phase CNS adaptation process: an initial phase enabling function with the transferred muscles, followed by a later phase abandoning this enabled function and restoring a control strategy that, while potentially less conflicted than the maladaptive state, resembled the original pattern, possibly representing a ‘good enough’ solution. These results highlight a multi-phase CNS adaptation process with distinct time constants in response to sudden bodily changes, offering potential insights into understanding and treating movement disorders.

## Introduction

The central nervous system (CNS) continuously adapts bodily functions in response to both external and internal challenges. Experimental models based on external perturbations, such as altered gravitational fields or distorted sensory feedback, have illuminated mechanisms of sensorimotor adaptation (Sugita, 1996; Davidson and Wolpert, 2003; Luauté et al., 2009; Fleury et al., 2019). Because the changes in the external environment can be controlled by the experimental design, these models provide an opportunity to assess how the CNS adapts to them. However, the transient and predictable nature of these changes may not fully capture the demands posed by internal, long-lasting, and unpredictable alterations to the body’s internal environment.

In contrast, the internal changes such as developmental growth (Power and Schlaggar, 2017), fatigue (Green, 1997), postural sway (Zemková, 2022), and sensory disorientation (Schärli et al., 2024) impose distinct challenges. Because identifying the source, extent, and time constant of changes in the internal environment is usually difficult, assessing the corresponding CNS adaptation is also challenging. Particularly, structural alterations to the musculoskeletal system, whether due to injury, disease, or surgery, fundamentally change the body’s biomechanics and sensorimotor associations, but the quantification of these changes is usually difficult. Accordingly, the way CNS remaps its motor control strategies corresponding to the changes is not yet well understood (Walker et al., 2004), although understanding such adaptations is crucial for elucidating the pathophysiology of motor impairments observed in chronic musculoskeletal conditions such as osteoarthritis, a degenerative joint disease (Hunter and Eckstein, 2009), and muscular dystrophy, which involves progressive muscle weakness (Mercuri and Muntoni, 2013).

To address this question, in this study, we employed a tendon transfer (TT) surgery model (Sperry, 1940), which introduces a controlled, sustained change to the musculoskeletal structure. TT surgery is a clinically well-established procedure (Gardenier et al., 2020) that surgically reattaches the tendon of a specific muscle to that of a surrounding one. This procedure relocates a specific muscle so that its contraction generates a new mechanical action and, consequently, novel somatosensory feedback. Because the internal change is controlled by the experimenter, the TT model provides a powerful platform to investigate how the CNS adapts to a new internal state. Another unique feature of the TT model is that it places permanent changes of the internal environment while leaving the CNS anatomically intact. Unlike CNS lesion models (Hoffmann et al., 2009), where the injury itself disrupts neural circuits and thereby complicates the assessment of the adaptive capacity of the CNS, TT offers a distinct advantage: it allows investigation of CNS-driven adaptation without the confounding effects of direct neural damage. TT has been used in various species, with studies suggesting different adaptive capacities, ranging from limited adaptation in adult rodents (Sperry, 1940; Sperry, 1942; Slawinska and Kasicki, 2002; Bowlus et al., 2003) to more substantial adjustments in cats (Yumiya et al., 1979; Loeb, 1999) and primates, including humans (Lee and Seung, 1999; Wester et al., 2018; Gaetz et al., 2023). Therefore, adaptability to TT appears enhanced in primates and humans. Altogether, this approach provides a controlled platform to examine how the CNS adapts to musculoskeletal changes in primates.

According to earlier reports, there are two motor control strategies that could be involved when the CNS needs to face the sensorimotor remapping posed by structural alterations to the musculoskeletal system. First, the CNS may employ modular building blocks such as muscle synergies, coordinated activations of muscle groups that reduce the dimensionality of motor control (d’Avella et al., 2003; Bizzi and Cheung, 2013). Second, skilled behaviors like fine finger movements often require fractionation, the capacity to activate muscles independently. When confronted with structural changes to the musculoskeletal system, does the CNS adapt by modulating existing synergies, or by shifting toward more fractionated control strategies? Addressing this question requires examining the evolution of neural control strategies over extended periods of recovery to capture both immediate adjustments and long-term learning.

This study aimed to identify long-term adaptive mechanisms of the primate CNS following structural changes to the musculoskeletal system. We investigated whether the CNS adapts by modulating existing muscle synergies or by altering the fractionated control of muscles specifically affected by the surgical alteration. We surgically altered the limb structure by performing crossed TTs of finger extensor and flexor muscle tendons. Using a trained finger-grasping task as our behavioral readout, we examined how the CNS recalibrates muscle activity to regain skilled motor function. Our findings provide new insights into the dynamic reorganization of motor control following structural changes of the body.

## Results

**Figure 1.**
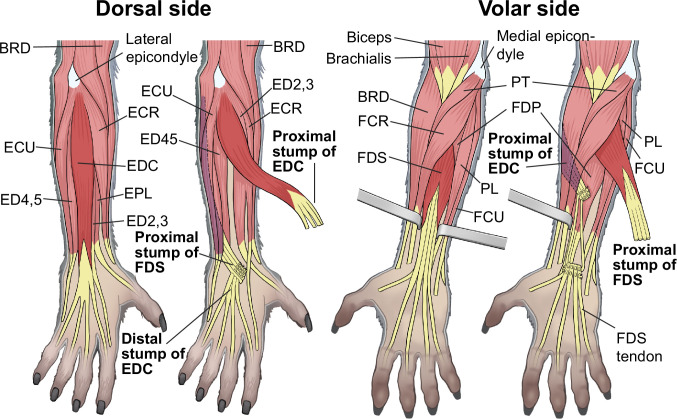
Muscle anatomy of the macaque forearm and the tendon transfer procedure. A schematic of the primary forearm muscles involved in the study, showing both the dorsal and volar views. The diagram illustrates the surgical crossed tendon transfer of the extensor digitorum communis (EDC) and flexor digitorum superficialis (FDS) tendons. All labeled muscles were implanted with electromyography (EMG) electrodes. Muscle abbreviations: BRD: brachioradialis, ECR: extensor carpi radialis, ECU: extensor carpi ulnaris, ED2,3: extensor digitorum-2,3, ED4,5: extensor digitorum-4,5, EPL: extensor pollicis longus (not implanted), FCR: flexor carpi radialis, FCU: flexor carpi ulnaris, FDP: flexor digitorum profundus, PL: palmaris longus, PT: pronator teres (not implanted). (The deltoid [DEL] muscle was also implanted in Monkey B but is not shown as it is a shoulder muscle.) See also Supplementary file 1A for a complete list of all muscle abbreviations, their full names, and their assigned synergies.

**Figure 2.**
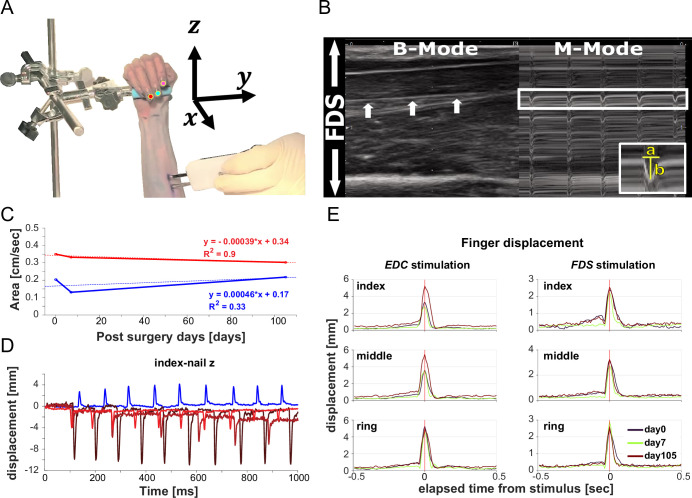
Long-term confirmation of tendon surgery effectiveness to alter mechanical properties. (**A**) Setup for the ultrasound measurement and video recordings of the stimulation-induced movements of the extensor digitorum communis (EDC) and flexor digitorum superficialis (FDS) tendons. (**B**) Sonogram of the FDS muscle and its intramuscular tendons. Left side (B-mode, i.e. brightness mode) shows the still image of the monkeys’ forearm at a given point in time. Right side (M-mode, i.e. motion mode) shows the staggered images of the FDS tendon displacement induced by muscle stimulation (50 mA). White arrows demarcate the FDS tendon which was used for the measurement. Grayscale gradations correspond to tissue densities: hyperechoic regions (white) denote denser structures like the surface of bones and tendons, while hypoechoic areas (black) signify less dense tissues such as adipose tissue and musculature. Inset demonstrates the area measurement. The area of the displacement waves was measured in the M-mode, representing the strength of muscle contraction. We measured the duration (a, s) and amplitude (b, cm) of three waves and calculated the average. Area = a*b/2(cm/s) for days 0, 7, and 105 after tendon transfer (TT). (**C**) Areas under the wave measured in the M-mode for 3 experimental days (0, 7, and 105 days post-TT) and regression lines in red and blue for FDS and EDC, respectively. R^2^>0.5 for FDS. The data suggested that muscle contractions induced by direct electrical stimulation were nearly constant. (**D**) Markers placed on the index, middle, and ring finger nails (**A**) were used to measure finger displacement in xyz-dimensions. We calculated the sum of the Euclidean distances of each marker from the origin of the 3D coordinate system as a scalar quantity. Observing the movement along the z-axis, it became reversed post-surgery, indicating a reversal from finger flexion to extension due to tendon transfer (D, blue = pre-TT at surgery day; dark brown = post-TT at surgery day; light brown = 1 week post-TT; red = 3 weeks post-TT). The scalar quantity of the fingers during muscle stimulation did not change much at day 0, 7, and 105 days (**E**), suggesting that there was no tendon rupture or slackening of the tendons postoperatively (EDC stimulation, left; FDS stimulation, right). Data were collected in Monkey A.

**Figure 3.**
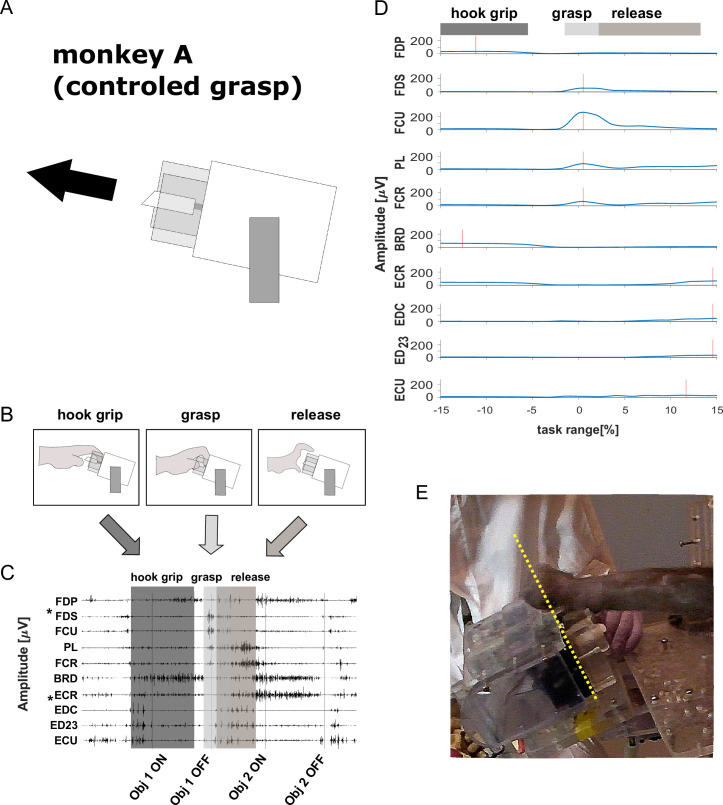
Experimental setup, task sequence, and typical electromyography (EMG) (Monkey A). (**A**) Schematic of the task object using a rod requiring Monkey A to perform a controlled grasp. (**B**) Schematic of the behavioral sequence (hook → grasp → release). (**C**) Typical EMG traces of a control session (high-pass filtered) for all recorded muscles. Gray boxes represent the task sequence. Obj 1 ON: start of the hold period of object 1. Obj 1 OFF: end of the hold period of object 1, i.e., object release. Obj 2 ON: start of the hold period of object 2. Obj 2 OFF: end of the hold period of object 2, i.e., object release. Tendons of the muscles marked with * were cross-transferred. (**D**) Rectified and smoothed EMG for all recorded muscles (average for one recording session; amplitude [μV] over task sequence [%]). Horizontal bars illustrate the corresponding behavioral periods; red vertical lines indicate peak amplitude for each muscle. (**E**) The time the monkey spent on the left side of the yellow dotted line while moving from object 1 to object 2 was measured and used to quantify the maladaptive behavior.

**Figure 4.**
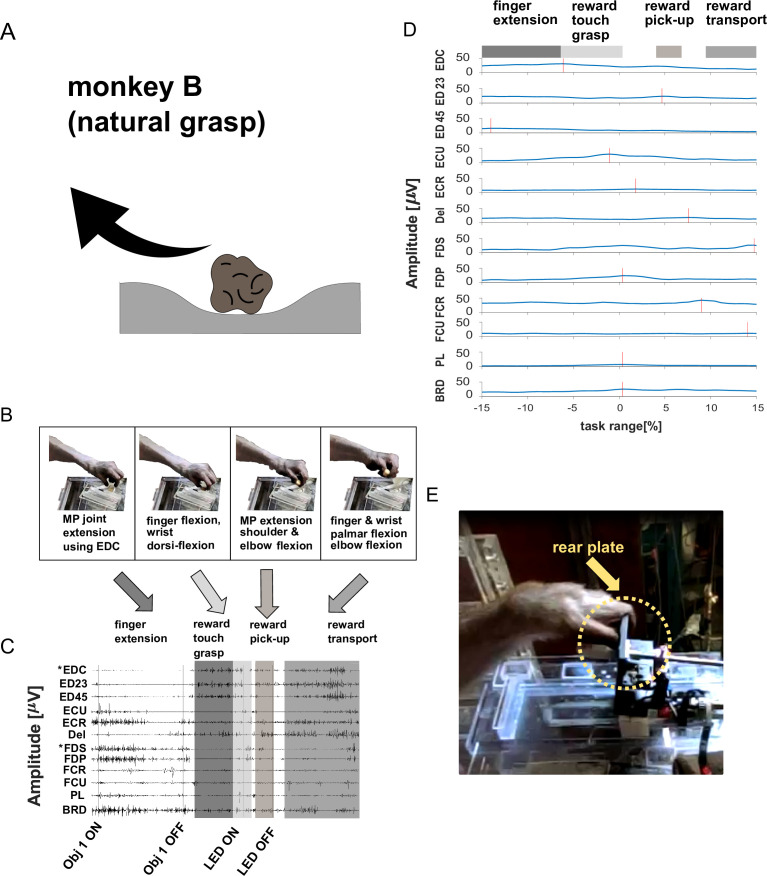
Experimental setup, task sequence, and typical electromyography (EMG) (Monkey B). (**A**) Schematic of the task requiring Monkey B to pick up food from a groove allowing for a more natural grasp. (**B**) Schematic of the task sequence (picking up food). (**C**) Typical EMG traces of a control session (high-pass filtered) for all recorded muscles. Gray boxes represent the task. Obj 1 ON: start of the hold period of object 1. Obj 1 OFF: end of object 1’s hold period, i.e., object release. LED ON: approximate start of the food touch. LED OFF: approximate time of food retrieval. Tendons of the muscles marked with * were cross-transferred. (**D**) Rectified and smoothed EMG for all recorded muscles (average for one recording session; amplitude [μV] over task sequence [%]). Horizontal bars illustrate the corresponding behavioral periods; red vertical lines indicate peak amplitude for each muscle. (**E**) Example of maladaptive behavior in Monkey B. The time the monkey spent in contact with or behind the object plate was measured and used to quantify the maladaptive behavior.

In this study, we developed a novel crossed TT animal model. This involved surgically swapping the tendons of two antagonistic finger muscles in a macaque monkeys’ forearm (Figure 1; a complete list of all muscle abbreviations, their full names, and their assigned synergies for each monkey is provided in Supplementary file 1A). The effectiveness and consistency of the surgery was confirmed by measuring: (i) the distance traveled by muscle fibers and their intramuscular tendons in the transferred muscle (Figure 2B and C) and (ii) the amount of fingertip displacement (Figure 2D and E). Both measurements were induced by percutaneous electrical stimulation over the transferred muscles (Figure 2A). For a detailed description of the results of these measurements, see the Methods section. The two monkeys were assigned slightly different tasks, allowing us to examine controlled grasping in Monkey A and a more natural grasp in Monkey B (refer to the Methods section and Figures 3 and 4 for further details). The two monkeys ultimately performed related but distinct grasping tasks, a methodological divergence that provided a valuable opportunity to test the generality of the core adaptive mechanisms. Monkey A performed a controlled grasping task requiring a fine precision grip, designed to study adaptation of fine motor control (Figure 3). While the same task was initially planned for both animals, Monkey B performed this task inconsistently, frequently varying its grip strategy, and we could not reinforce this monkey to perform a single strategy consistently.

Therefore, to ensure reliable task engagement and in accordance with the ethical principle of Reduction and Refinement (the ‘3Rs’; Tannenbaum and Bennett, 2015), the task for Monkey B was modified to a more naturalistic food retrieval grasp that the animal performed consistently (Figure 4).

Once the monkeys had mastered their respective tasks, we recorded control sessions and then performed the crossed TT. Post-surgery, and once the monkeys had fully recovered and were able to perform the task independently (~3–4 weeks), electromyography (EMG) recordings and behavior were resumed (Videos 1–7).

**Video 1.** Control behavior (Monkey A – pre-tendon transfer [TT]). This video demonstrates Monkey A’s baseline performance on the reaching task before the crossed TT procedure. It illustrates the typical, coordinated movements exhibited by the monkey in its unaltered state, serving as a control for comparison with post-surgical behavior. Observe the smooth and accurate reaching and grasping motions as the monkey performs the task.

**Video 2.** 29 days post crossed tendon transfer (Monkey A). This video documents Monkey A’s attempts to perform the reaching task 29 days after undergoing the crossed tendon transfer procedure. At this early-stage post-surgery, the video clearly shows the significant impact of the procedure on the monkey’s motor control. Observe the marked malcoordination in the monkey’s reaching movements. The reaching attempts are furthermore characterized by ‘explorative’ finger movements over the object. The monkey’s reliance on the experimenter for support highlights the difficulty it experiences in performing the task independently.

**Video 3.** 42 days post crossed tendon transfer (Monkey A). This video shows Monkey A’s progress 42 days post-surgery. While an improvement in motor control is evident compared to the 29-day mark, the monkey still exhibits some residual deficits. Observe the monkey’s attempts to perform the task autonomously. Although it can now perform the task fully without experimenter assistance, it continues to rely on the support of its unaffected arm, suggesting ongoing challenges with coordination and strength.

**Video 4.** 100 days post crossed tendon transfer (Monkey A). This video demonstrates Monkey A’s performance 100 days after the crossed tendon transfer. At this point, the monkey has achieved substantial recovery and performs the reaching task with near-normal proficiency. Observe the smooth, coordinated movements, and the monkey’s ability to execute the task independently and accurately. This footage showcases the significant recovery of motor function following the procedure.

**Video 5.** Control behavior (Monkey B – pre-tendon transfer [TT]). This video establishes the baseline performance for Monkey B prior to the crossed TT procedure. It shows the monkey’s typical, coordinated reaching behavior in its unaltered state, providing a control for comparison with its post-surgical performance. Observe the precision and fluidity of the monkey’s movements as it executes the task.

**Video 6.** 29 days post crossed tendon transfer (Monkey B). This video documents Monkey B’s performance 29 days after the crossed tendon transfer. Like Monkey A, Monkey B exhibits significant motor deficits. The video clearly demonstrates the malcoordinated reaching, characterized by overshooting the target and bumping into objects.

**Video 7.** 68 days post crossed tendon transfer (Monkey B). This video shows Monkey B’s recovery 68 days post-surgery. At this time point, Monkey B has achieved a full recovery and performs the reaching task with accuracy and coordination comparable to its pre-surgical baseline. Observe the smooth and efficient movements, demonstrating the successful recovery of motor function.

### Functional recovery follows a biphasic trajectory

**Figure 5.**
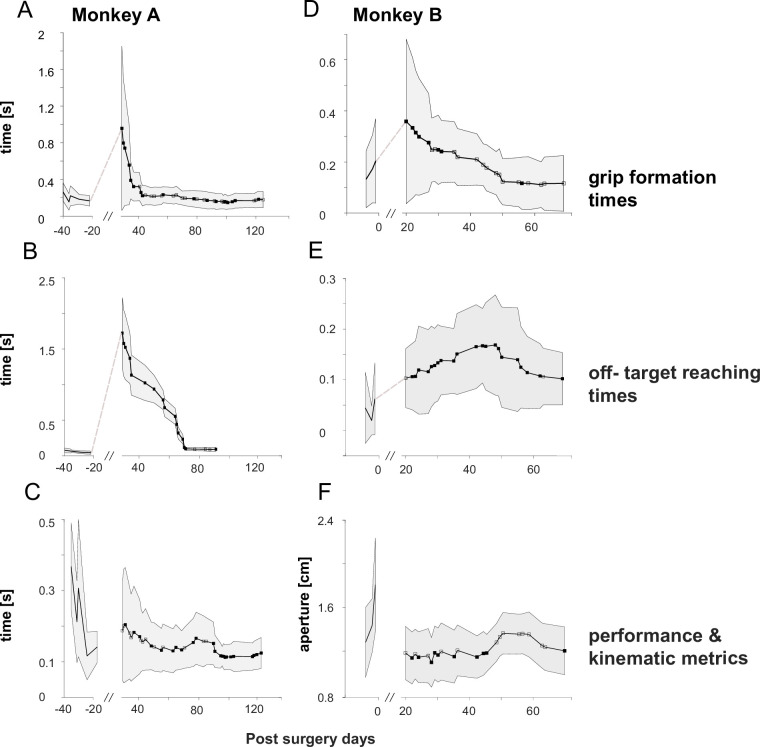
Behavioral and kinematic metrics of motor recovery. (**A, D**) Grip formation times (mean ± SD; n=20 trials) for Monkey A (**A**) and Monkey B (**D**). (**B, E**) Duration of off-target reaching movements (mean ± SD; n=10 trials) for Monkey A (**B**) and Monkey B (**E**). (**C**) Pull time duration for Monkey A. (**F**) Grasp aperture size for Monkey B. Filled squares indicate significant difference from pre-tendon transfer (TT) baseline (p<0.05, two-sample t-test). All data are plotted over days relative to TT.

Despite the now reversed roles of the transferred muscles, the monkeys were able to recover their grasping performance within 2 months, assessed by both the return of key behavioral metrics to pre-surgical levels (Figure 5A and D) and their consistent ability to perform the task independently (Videos 3, 4, and 7). However, this recovery was not immediate. The monkeys spent a period of 1–2 weeks in their home cages for recovery, followed by several weeks of assisted task practice. Formal recordings began once the monkeys could perform the task consistently without assistance. The initial post-surgical period was defined by a phase of significant motor impairment, primarily characterized by off-target reaching trajectories (Figure 5B and E) and prolonged grip formation times (Figure 5A and D). These off-target reaching movements included inefficient, ‘explorative’ trajectories in Monkey A and target overshoots in Monkey B (Videos 2, 3, and 6). This recovery process was quantified across several behavioral and kinematic metrics (Figure 5). A comparison of pre-TT behavioral variability on the grip formation task (Figure 5D) revealed that Monkey B was substantially more variable (coefficient of variation [CV] = 81.93%) than Monkey A (CV = 46.62%). This difference in variance was highly statistically significant (Ansari-Bradley test, p<0.0001), confirming a difference in baseline stability between the subjects likely due to the less constrained nature of Monkey B’s task. Post-surgery, variability in grip formation time initially increased dramatically for Monkey A (early phase CV = 132.98%), while remaining high for Monkey B (early phase CV = 76.46%). In the late phase, Monkey A’s variability stabilized slightly above baseline (late phase CV = 54.79%), whereas Monkey B’s variability increased further, exceeding its pre-TT level (late phase CV = 96.59%), suggesting persistent inconsistency in grasp timing for this animal. Variability patterns differed for the other metrics: Monkey A’s pull time variability decreased below baseline in the mid and late phases (pre CV = 64.97%, mid CV = 41.30%, late CV = 43.01%), indicating a refinement of this action, while Monkey B’s grasp aperture variability remained consistently low throughout recovery (pre CV = 26.37%, early CV = 23.80%, mid CV = 19.78%, late CV = 19.35%). The initial post-surgical period was defined by motor performance that we classify as maladaptive due to the persistence of inefficient, off-target reaching, and prolonged movement durations. First, task-related grip formation times were significantly longer immediately post-TT (Monkey A: 197.7±92.2 vs. 660.6±221 ms, p=0.014; Monkey B: 169.7±14 vs. 316.3±31.9 ms, p=0.016), taking approximately 40 days for Monkey A and 48 days for Monkey B to return to and stabilize at pre-surgical levels (Figure 5A and D). Second, the duration of off-target reaching was substantially elevated, stabilizing approximately after 70 days in Monkey A and 55 days in Monkey B (Figure 5B and E). Following this phase, motor performance showed a gradual recovery in efficiency (Figure 5C) and kinematics (Figure 5F). Ultimately, overcoming these initial maladaptive behaviors through the gradual refinement of motor performance led to the stabilization of grasping by the end of the experimental period.

### A two-phase adaptation is observed in the EMG activity of individual muscles

**Figure 6.**
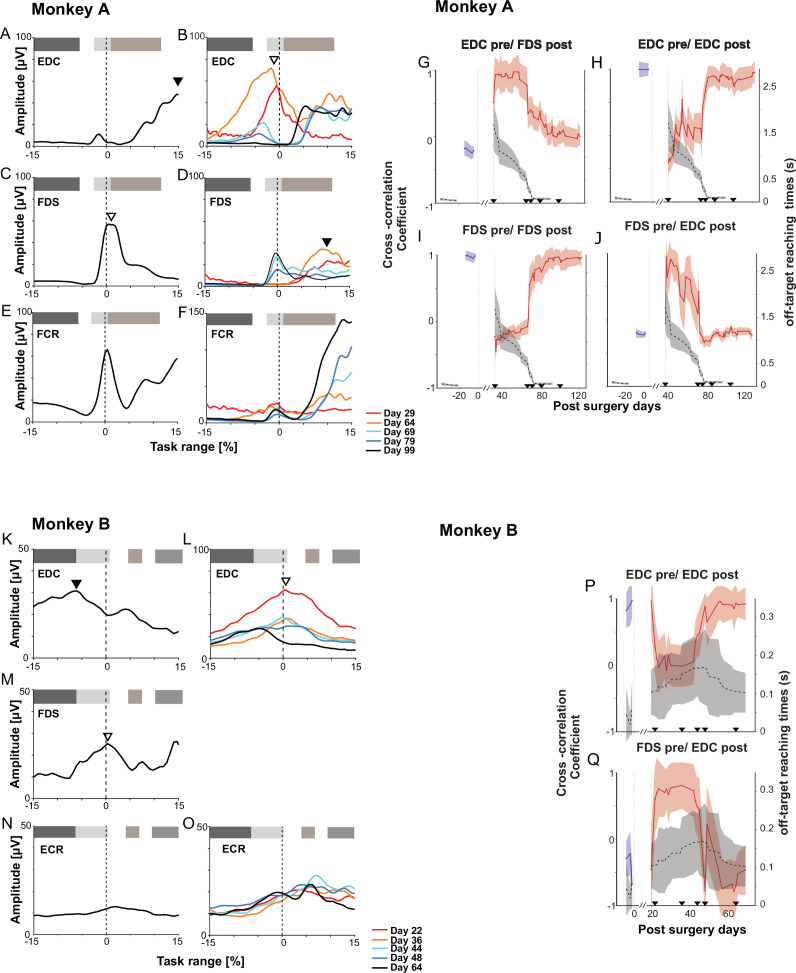
Temporal electromyography (EMG) profiles and cross-correlation analysis. Temporal EMG profiles for Monkey A (left, **A–J**) and Monkey B (right, **K–Q**). (**A–F, K–O**) Average EMG activity profiles aligned to task events (0%; object release for A, food touch for B). Shaded envelopes represent standard deviations. Triangles indicate peak activity during extension (▼) or flexion (▽). Colored traces denote post-surgery landmark days. (**A–D, K–M**) Comparison of transferred muscles. Note the temporal shift in the post-surgery profile (**B, L**) relative to the pre-tendon transfer (TT) baseline (dashed lines). (**E–F, N–O**) Profiles of non-transferred muscles. (**G–J, P–Q**) Zero-lag cross-correlation coefficients between post-surgery EMG profiles and the pre-TT baseline profiles plotted over time. (**H, I, P**) Correlation coefficients calculated against the muscle’s *own* pre-TT baseline. (**G, J, Q**) Correlation coefficients calculated against the *antagonist’s* pre-TT baseline (e.g. post-EDC vs. pre-FDS). Black dashed lines on the right y-axis indicate behavioral error metrics (off-target reaching time for Monkey A; contact duration for Monkey B; gray shading represents SD). The // represents the recovery period. EDC, extensor digitorum communis; FDS, flexor digitorum superficialis.

Initially, we investigated whether EMG activity exhibited any changes post-TT. Given that we interchanged the tendons of an antagonistic muscle pair, functionally rendering the flexor digitorum superficialis (FDS) an extensor and the extensor digitorum communis (EDC) a flexor (as confirmed mechanically, Figure 2), a biomechanically sound adaptation would be to use the former finger extensor (EDC) for finger flexion, and the former finger flexor (FDS) for finger extension during a grasping task. We therefore analyzed EMG activity from both transferred and non-transferred muscles to determine if these expected functional changes occurred. Figure 6 shows a comparison of EMG activity profiles for the two transferred muscles, EDC and FDS, in Monkey A (Figure 6A–D). Prior to surgery, the control showed distinct and contrasting EMG profiles in both muscles. For instance, peak activity in EDC (▼) occurred long after completion of the grasping action (indicated by vertical dashed lines at 0% task range) at 15% task time (Figure 6A), coinciding with the animal pre-shaping its hand to prepare for the subsequent grasp, whereas peak activity in FDS (▽) was observed immediately after completion of the grasping action (0.49%; Figure 6C). The question we posed was whether the post-surgery activity of EDC resembled its original profile (without adaptation to surgery) or the activity of FDS (the anticipated profile following surgical adaptation), and vice versa. Our findings suggested that EMG activity patterns largely shifted in a manner consistent with the new mechanical function imposed by the transfer. Early after TT, peak activity of EDC occurred at –0.58% task time (Figure 6B; days 29 [red line] and 64 [orange]), which aligns with peak activity of FDS prior to TT (Figure 6C; black line). Also, for FDS, peak activity occurred at 11% task time (Figure 6D; ▼), which aligned with peak activity of EDC (Figure 6A).

**Figure 6—figure supplement 1.**
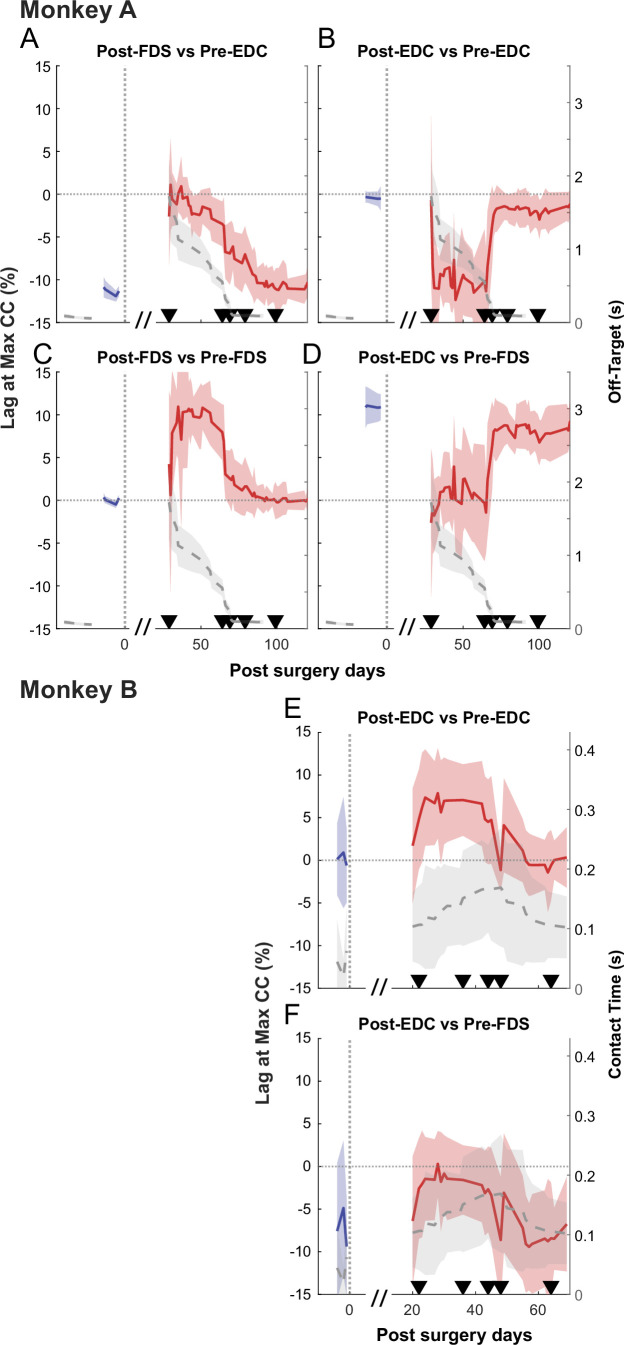
Evolution of time lag at peak cross-correlation between transferred muscle electromyographies (EMGs). Quantification of the optimal time lag (in % task range) yielding the maximum cross-correlation between individual muscle EMG profiles. (**A–D**) Monkey A; (**E–F**) Monkey B. Each panel plots the time lag calculated between the recorded EMG profile and the average pre-tendon transfer (TT) baseline (specific comparisons, e.g. ‘post-EDC vs. pre-EDC’, are indicated in titles). Left y-axis: time lag. Blue traces: pre-surgery data; red traces: post-surgery data. Right y-axis (gray dashed traces): behavioral error metrics (off-target reaching duration for Monkey A; contact time for Monkey B). A positive lag value indicates that the muscle activity is delayed relative to the pre-surgery baseline; a negative value indicates it is advanced (occurs earlier). The dotted horizontal line indicates zero lag. Shaded envelopes represent standard deviations. Black triangles indicate landmark days. EDC, extensor digitorum communis.

**Figure 6—figure supplement 2.**
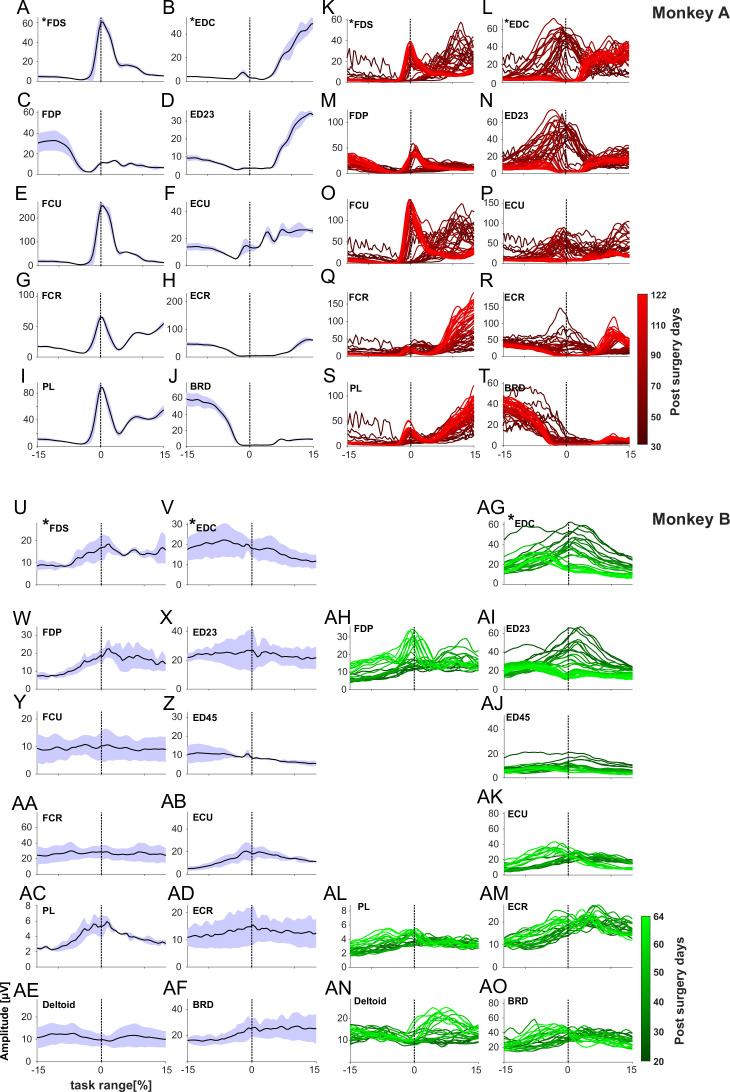
Electromyography (EMG) activity profiles for all recorded muscles across all sessions. EMG activity profiles [μV] for all recorded muscles in Monkey A (**A–T**) and Monkey B (**U–AO**). (**A–J, U–AF**) Average pre-tendon transfer (TT) profiles (± SD in blue). (**K–T, AG–AO**) Post-surgery profiles for each recording day, aligned on object release (Monkey A) or food touch (Monkey B). Lighter colors indicate later recording days (see color bar). Muscles with surgically transferred tendons are marked with an asterisk (*).

**Figure 6—figure supplement 3.**
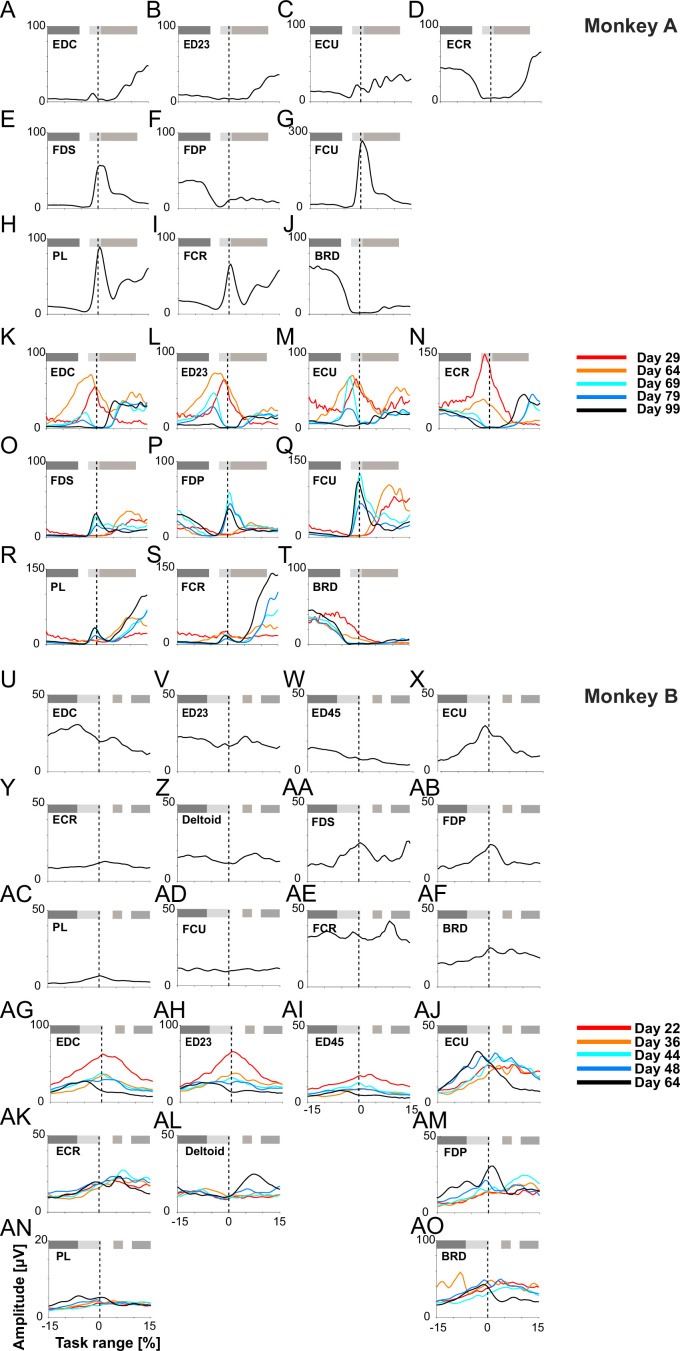
Electromyography (EMG) activity profiles of ‘landmark days’. Monkey A: EMG activity profiles [μV] of selected ‘landmark days’ for all recorded muscles (pre- and post-tendon transfer [TT], A–J and K–T, respectively). Profiles are aligned on hold off-set (object 1) at zero percent task range [%] (dashed lines). Horizontal gray bars mark the three behavioral periods (hook, grasp, and release, respectively). Monkey B: pre- and post-TT, U–AF and AG–AO, respectively. Profiles are aligned on LED on-set (food touch) at zero percent task range [%] (dashed lines). Horizontal gray bars mark the four behavioral periods (finger extension, food touch, food pick-up, and food transport, respectively).

**Figure 6—figure supplement 4.**
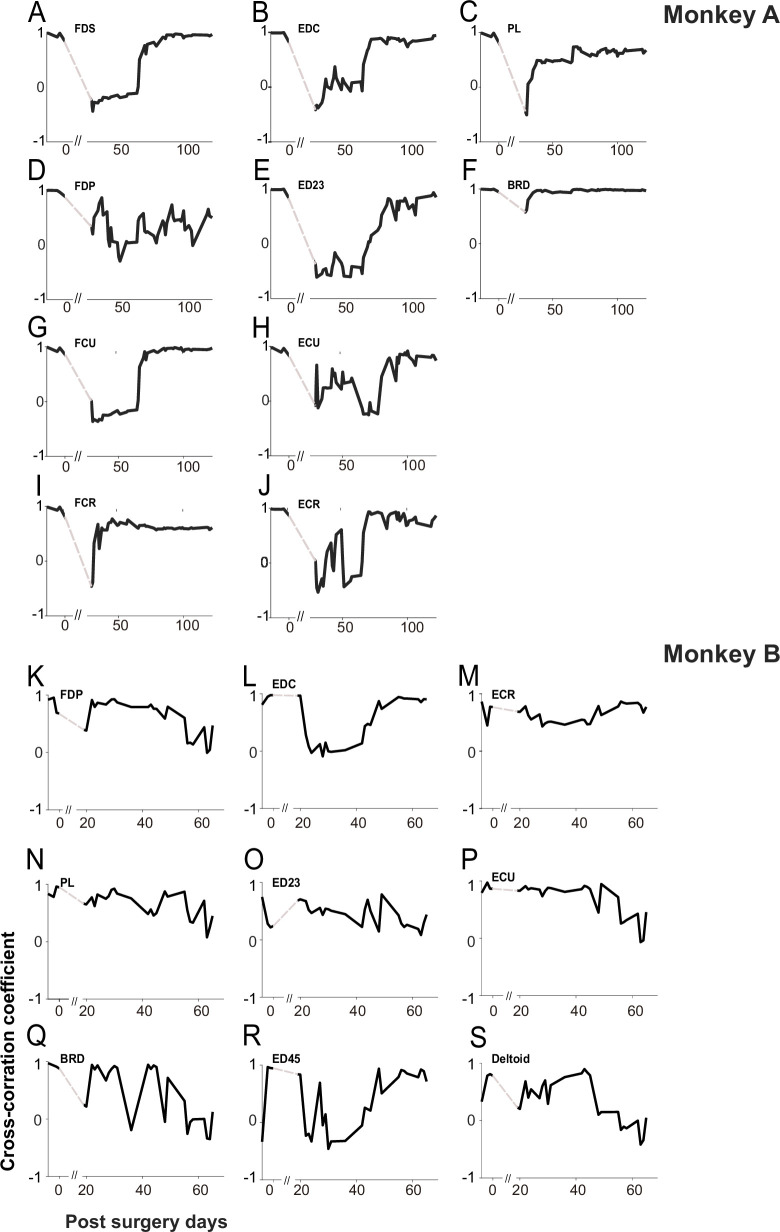
Cross-correlation analysis for individual muscles. Cross-correlation of each muscle’s electromyography (EMG) activity profile against its own pre-tendon transfer (TT) control data. The plots show the evolution of the correlation coefficient over post-surgery days for Monkey A (**A–J**) and Monkey B (**K–S**), quantitatively illustrating the time course of adaptation for each muscle.

This was corroborated by the results in Monkey B (Figure 6, panels K–M). Although the EMG patterns of both muscles varied significantly as the two monkeys performed different types of grasping actions, we found that the EMG of EDC peaked at –6.12% before (Figure 6K; ▼) and 0.35% and 1.05% after TT (Figure 6L; ▽, days 22 and 36, respectively), closely matching the timing for FDS control data (Figure 6M). The corresponding analysis for FDS could not be performed in Monkey B, since the EMG signal of FDS was lost early after TT (see Methods for details).

We were able to extend our recordings to 122 and 64 days post-TT for Monkeys A and B, respectively. This allowed us to examine whether the adaptations observed in the early period were consistent throughout the experimental period. These additional days (Figure 6B and D; 69 [cyan], 79 [blue], and 99 [black] post-TT; see Figure 6—figure supplement 1 for recordings from all days) showed that the EMG activity profiles for both muscles unexpectedly returned to their pre-TT state. For example, as already described, the EMG signal of EDC at days 29 and 64 shared the characteristics of FDS, but by the following day (only 5 days later, day 69 [light blue] post-TT) already exhibited pre-TT characteristics of EDC, continuing as such until the end of the recording period (day 79 [blue] and 99 [black]). This was confirmed for the EDC muscle of Monkey B (Figure 6K and L; Figure 6—figure supplement 1U–AO for recordings from all days).

To relate these neural changes to functional recovery, we overlaid the behavioral error metric (off-target reaching time; gray dashed lines) onto the cross-correlation plots (Figure 6G–J). Figure 6H (red line) illustrates cross-correlation coefficients between the EDC EMG profile post-TT (EDC-post) and the original EDC control data (EDC-pre) in Monkey A. Figure 6I shows coefficients for EDC-post and FDS-pre correlations in the same monkey. The coefficient corresponding to the original profile decreased to between –0.3 and 0.2 over approximately 65 days (Figure 6H). In contrast, the coefficient corresponding to the expected EMG profile exhibited an inverse pattern, with low coefficients pre-TT and a high coefficient (of 0.9) post-TT (Figure 6J). Cross-correlation analysis for FDS (Figure 6G and I) revealed a similar pattern. Cross-correlation coefficients for FDS-pre- vs. FDS-post analysis declined from 1 to negative values, only to rebound to their original values within a month (day 79; Figure 6G). This outcome aligns with our previous observations (Figure 6B and D) and were corroborated by the results from Monkey B (Figure 6K–Q). Here, cross-correlation coefficients dropped from +0.95 to +0.1 for EDC-pre vs. EDC-post comparison, and increased from –0.5 to 0.9 for the FDS-pre vs. EDC-post comparison (Figure 6P and Q). After 42 days post-TT, cross-correlation coefficients started to gradually increase or decrease to +0.9 and –0.7, respectively. This indicates that the anticipated initial changes in EMG activity profiles are not permanent. Instead, they revert to their original patterns 2 months post-TT. A supplementary analysis of the time lag at peak correlation (Figure 6—figure supplement 1) confirmed that the optimal lag for individual muscles also fluctuated significantly during the early adaptive phase before stabilizing, reflecting the process of temporal re-coordination of individual muscle commands. Interestingly, changes in EMG pattern were not isolated to the transferred muscles; non-transferred muscles exhibited a variety of complex adaptations (Figure 6, Figure 6—figure supplements 2–4). A comprehensive overview of the average EMG profiles for all recorded muscles across all recording sessions and landmark days is provided in Figure 6—figure supplement 2 (Monkey A) and Figure 6—figure supplement 3 (Monkey B), respectively. As shown in these figures, many agonists adapted in concert with their transferred synergist, following the same two-phase ‘swap-and-revert’ pattern seen in Figure 6. In Monkey A, these included extensors ED23 and ECU, while in Monkey B, they included ED23, ED45, and ECU (see Figure 6—figure supplement 2 for details). In contrast, other muscles showed patterns that were incompatible with a simple swap. For example, the non-transferred flexor carpi radialis (FCR) in Monkey A showed a distinct adaptive profile characterized by a drastic initial decrease in one activity peak and a consistent increase in a later peak (Figure 6E and F). A similar incompatible pattern was seen in palmaris longus (PL) (Figure 6—figure supplement 1H, I, R, S). Finally, some muscles, like the extensor carpi radialis (ECR) in Monkey B, remained relatively stable post-surgery (Figure 6N and O). These diverse patterns in non-transferred muscles do not necessarily contradict a modular control strategy (d’Avella et al., 2003; Bizzi and Cheung, 2013); rather, they likely reflect the fact that different muscles are primary members of different synergies (i.e. they possess the highest weightings within specific modules; e.g. FCR and PL in Synergy C), each undergoing its own task-specific adaptation.

### Adaptation occurs through modulating the activation of stable muscle synergies

**Figure 7.**
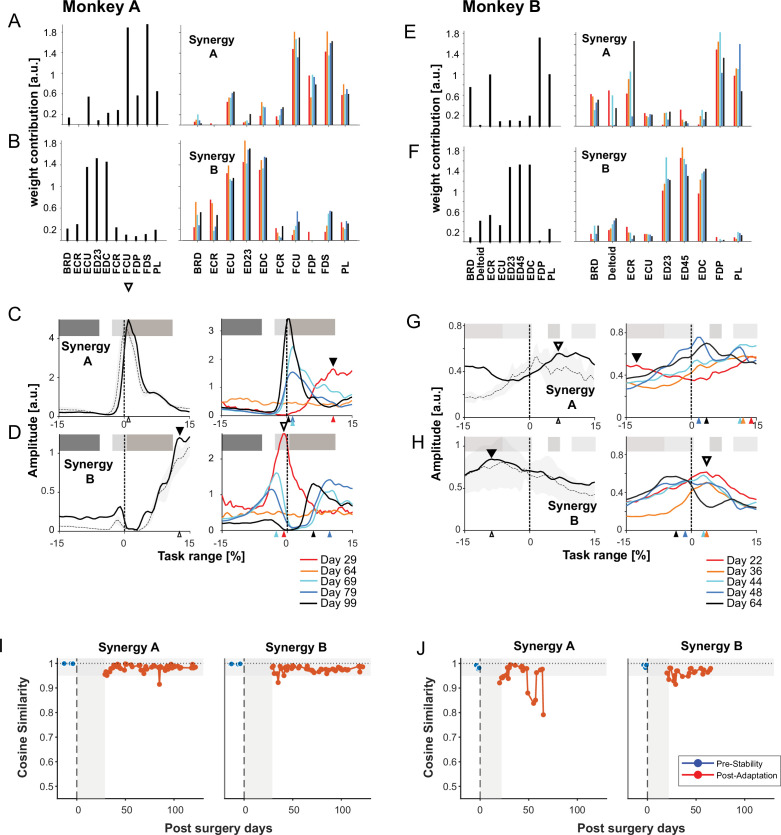
Spatial structure and temporal activation of primary synergies. Analysis of the two primary synergies: Synergy A (flexor) and Synergy B (extensor). (**A, B, E, F**) Spatial synergy weights (W) showing the contribution of each muscle to the synergy. Bar plots represent the average weights across all recording days. (**C, D, G, H**) Temporal activation profiles (**C**) aligned to task events (0%). Dashed lines with shaded tubes indicate the average pre-tendon transfer (TT) electromyography (EMG) profiles of the key contributing muscles (flexor digitorum superficialis [FDS], extensor digitorum communis [EDC], flexor digitorum profundus [FDP]) for visual comparison with the synergy profile. Symbols and alignment are as described in Figure 6. (**I, J**) Quantification of spatial stability. Cosine similarity of spatial synergy weights (W) calculated between individual recording days and the pre-TT average. Blue markers indicate pre-TT control days; red markers indicate post-TT days. The horizontal gray shaded region (0.95–1.0) denotes the range of high baseline stability.

To determine whether the CNS adapted by fractionating muscle control or modulating existing modules, we extracted muscle synergies using non-negative matrix factorization (NMF) (Lee and Seung, 1999). Four synergies accounted for >80% (Cheung et al., 2012) of the variance in both monkeys (see Figure 7—figure supplement 1 for VAF plots; >85% for both monkeys; the results of the original and shuffled datasets are shown. See also the synergy weights [W] and activation profiles [C] for both monkeys and all synergies in Figure 7—figure supplements 2 and 3, respectively). We first focused on the two primary synergies responsible for the main task axis: the finger flexor synergy (Synergy A) and the finger extensor synergy (Synergy B) (Figure 7).

**Figure 7—figure supplement 1.**
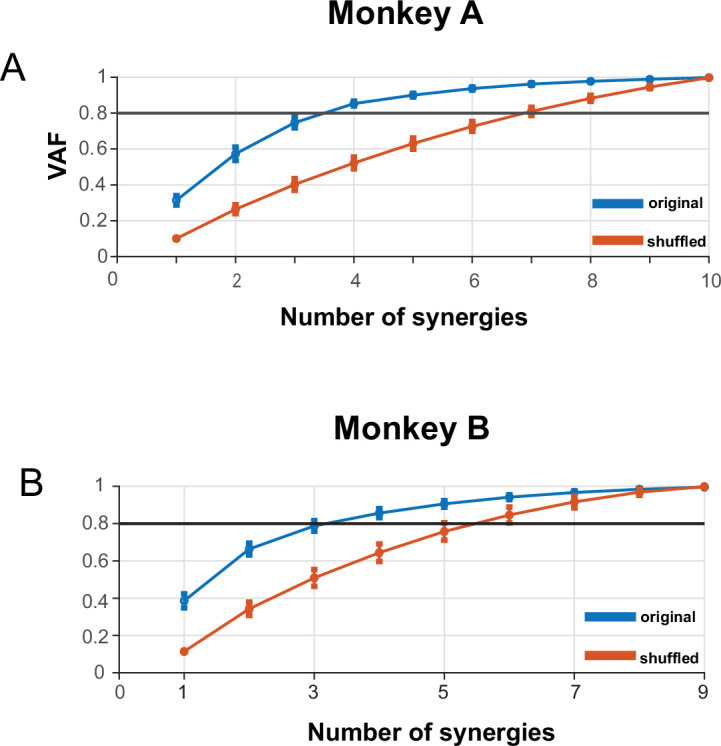
5 Variance accounted for (VAF). (**A–B**) Cumulative VAF plotted against the number of synergies for Monkey A (bottom) and Monkey B (top). Blue lines: original data; red lines: shuffled data. The black horizontal line indicates the 80% threshold used to determine the number of synergies.

**Figure 7—figure supplement 2.**
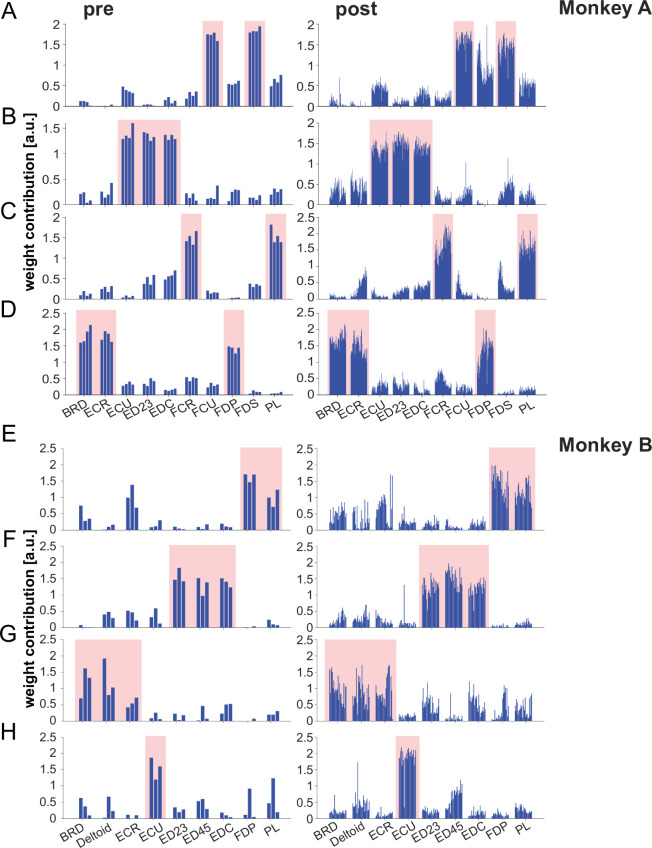
Synergy weights of all experimental sessions. (**A–D**, Monkey A) Synergy weights for each extracted muscle synergy. Each recording day was plotted individually before (left column) and after tendon transfer (TT) surgery (right column). Red boxes indicate the main contributing muscles to each of the corresponding synergy. (**E–F**, Monkey B). (**A, E**) Synergy A, (**B, F**) Synergy B, (**C, G**) Synergy C, and (**D, H**) Synergy D.

**Figure 7—figure supplement 3.**
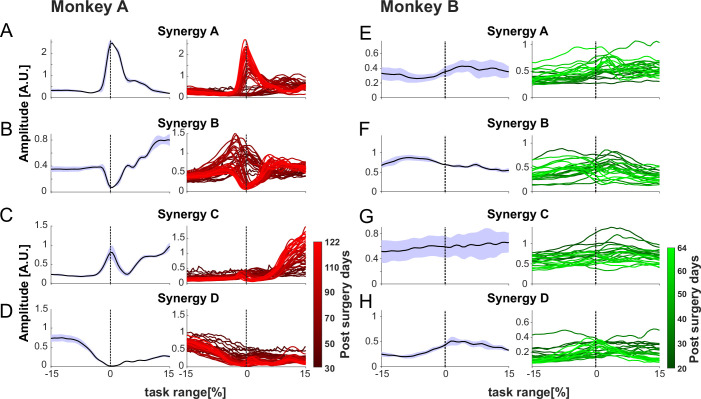
Time varying activation profiles of muscle synergies. Comparison of temporal activation profiles (y-axis: amplitude [a.u.]) for muscle synergies (**A**–**D**) between the pooled pre-surgery control period (blue traces; mean ± SD) and the final post-surgery recording day (red traces; mean ± SD). (Top row) Monkey A. (Bottom row) Monkey B. To identify specific phases where the recovered motor plan differed from baseline, a point-by-point Wilcoxon rank-sum test was performed (Bonferroni-corrected for multiple comparisons). Black markers at the top of each panel indicate time points where the activation amplitude remained statistically significantly different (p<0.05) from the pre-surgery baseline. The widespread presence of these significant differences, even where temporal shapes appear similar, confirms that the final motor state is a functional approximation (‘good enough’) rather than a perfect restoration of the original control strategy.

**Figure 7—figure supplement 4.**
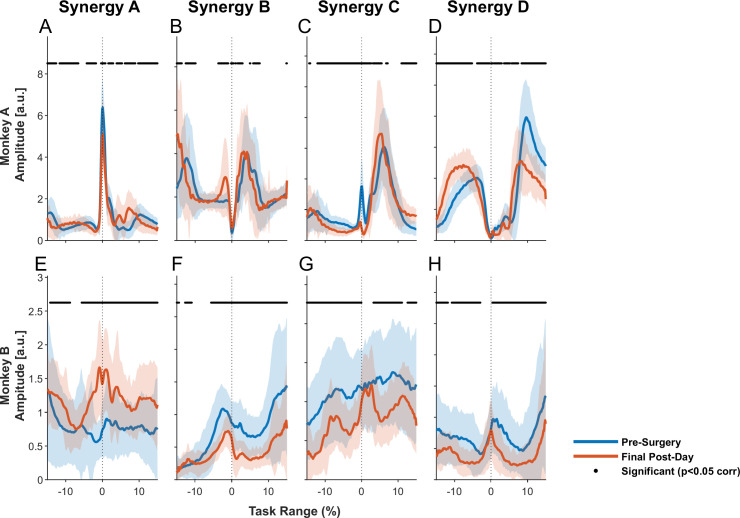
Quantitative comparison of pre-surgery and final post-surgery synergy activation profiles.

We first analyzed whether the spatial structure of these primary synergies changed. To distinguish genuine adaptation from natural variability, we established a baseline by calculating the cosine similarity of spatial synergy weights (W) across all pre-surgery days (Figure 7I and J, blue traces). This revealed remarkably high stability (>0.99). Post-surgery, the core structure remained conserved. The cosine distance between pre- and post-surgery weights for the same synergy pairs was low (Monkey A: 0.03±0.03; Monkey B: 0.09±0.11), whereas for different pairs it was high (>0.60). This confirms that the CNS maintained the stable, neurally constrained building blocks of the primary flexor and extensor modules despite the altered biomechanics.

In contrast to the stable spatial structure, the temporal activation profiles of these primary synergies underwent drastic changes. In Monkey A, the flexor Synergy A (originally peaking at +0.97% task time) and extensor Synergy B (originally peaking at 12.62%) swapped their timing profiles shortly after surgery (Figure 7C and D). Specifically, the extensor synergy shifted to peak during the flexion phase and vice versa. This ‘swap’ persisted during the early impairment phase before reverting toward the original timing in the late phase. A similar ‘swap-and-revert’ pattern was observed in Monkey B (Figure 7G and H), where the extensor Synergy B shifted from a pre-surgery peak at –8.75% to a post-surgery peak of +3.5% (matching the flexor phase) before recovering.

To formally quantify the quality of this reversion, we compared the pre-surgery activation profiles with those from the final day of recording. We found that while the temporal shape was highly preserved (cosine similarity>0.90; Supplementary file 1B), the profiles remained statistically distinct (permutation test, p<0.0001; Figure 7—figure supplement 4). This confirms that the recovered motor program represents a ‘good enough’ functional approximation rather than a perfect mathematical restoration of the baseline.

**Figure 8.**
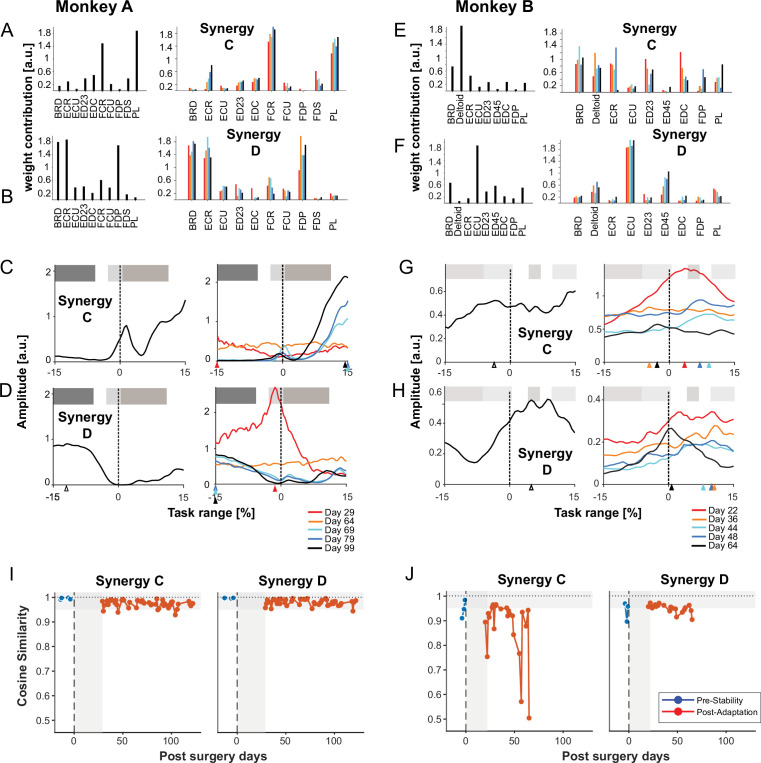
Analysis of secondary muscle synergies. (**A–H**) Analysis of secondary Synergies C (wrist flexor) and D (wrist extensor). (**A, B, E, F**) Spatial synergy weights (**W**) showing the contribution of each muscle. (**C, D, G, H**) Temporal activation profiles (**C**) aligned to task events (0%). Colored traces denote post-surgery landmark days. Layout and symbols are as described in Figure 6. (**I, J**) Quantification of spatial stability for Synergies C and D. Cosine similarity of spatial weights calculated between individual recording days and the pre-tendon transfer (TT) average. Blue markers indicate pre-TT control days; red markers indicate post-TT days. The horizontal gray shaded region (0.95–1.0) denotes the range of high baseline stability.

We next examined the secondary synergies involved in wrist control and stabilization (Figure 8). In contrast to the coherent ‘swap’ seen in the primary synergies, these modules exhibited distinct, task-specific adaptations.

In Monkey A, Synergy C (a wrist flexor synergy involving PL and FCR) showed a temporal shift that was incompatible with a simple swap (Figure 8C). While its spatial weights remained stable (Figure 8A and I), its activation peak shifted from the post-grasp phase (+1.46%) to the pre-grasp phase, increasing in magnitude over time. This pattern corresponds to the recruitment of wrist flexors to enable the compensatory tenodesis grasp. Synergy D (wrist extensor) underwent a single notable change on day 29 but quickly reverted (Figure 8D). In Monkey B, Synergy C showed no discernible trend (Figure 8G), while Synergy D exhibited a complex pattern of immediate change followed by a gradual shift in the late adaptation period (Figure 8H). Notably, the activation amplitude of Synergy D remained significantly suppressed compared to baseline (Figure 8H; Figure 7—figure supplement 2H). This suppression of the wrist extensor likely reflects the specific biomechanical requirements of the tenodesis strategy employed by Monkey B: relaxing the wrist extensors facilitates the necessary wrist flexion for hand opening. These independent changes in secondary synergies likely served to reinforce the overall functional recovery driven by the primary modules.

**Figure 9.**
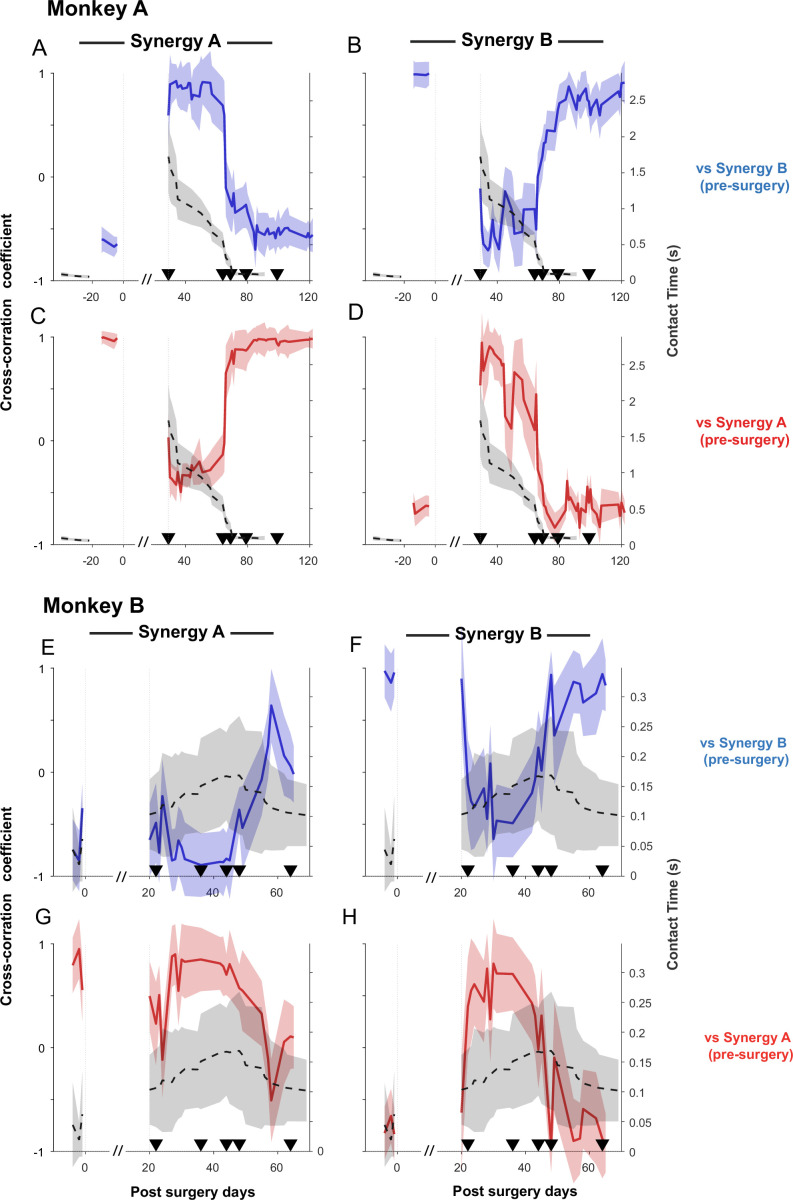
Cross-correlation analysis of primary synergy activation. Zero-lag cross-correlation coefficients plotted over post-surgery days for Monkey A (**A–D**) and Monkey B (**E–H**). Activation patterns of the primary flexor (Synergy A) and primary extensor (Synergy B) were cross-correlated with pre-tendon transfer (TT) baseline profiles. (Top row: **A**, **B, E, F**) Correlations calculated against the pre-TT *extensor* synergy (Synergy B, blue traces). (Bottom row: **C**, **D, G, H**) Correlations calculated against the pre-TT *flexor* synergy (Synergy A, red traces). (**C, G**) Correlation of Synergy A with its own pre-TT baseline. (**B, F**) Correlation of Synergy B with its own pre-TT baseline. (**A, E, D, H**) Cross-correlations between antagonistic synergies (e.g. A is Synergy A vs. pre-Synergy B). Black dashed lines on the right y-axis indicate behavioral error metrics (gray shading represents SD). The // represents the recovery period. Triangles indicate landmark days.

**Figure 10.**
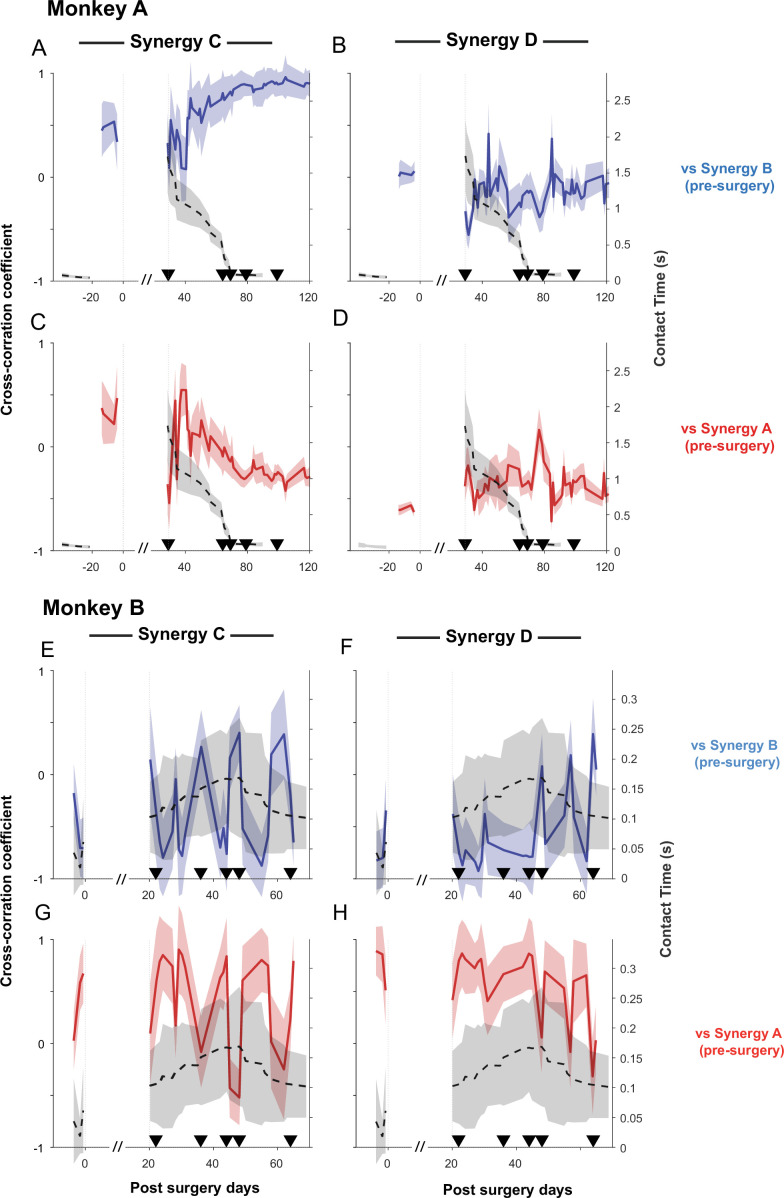
Cross-correlation analysis of secondary synergy activation. Zero-lag cross-correlation coefficients for the secondary synergies (**C and D**) plotted over post-surgery days for Monkey A (**A–D**) and Monkey B (**E–H**). Activation patterns were cross-correlated with the pre-tendon transfer (TT) profiles of the primary synergies to assess changing affiliations. (Top row: **A**, **B, E, F**) Correlations calculated against the pre-TT *extensor* synergy (Synergy B, blue traces). (Bottom row: **C**, **D, G, H**) Correlations calculated against the pre-TT *flexor* synergy (Synergy A, red traces). (Left columns: **A**, **C, E, G**) Synergy C correlations. (Right columns: **B**, **D, F, H**) Synergy D correlations. Black dashed lines on the right y-axis indicate behavioral error metrics.

Again, the characteristics of these changes in activation profiles of muscle synergies were also quantitatively confirmed by cross-correlation analysis of all four muscle synergies in both monkeys. The coefficients were plotted over the course of experimental days in relation to tendon surgery together with the overlaid behavioral error metric (off-target reaching time; gray dashed lines) (Figure 9). In this analysis, the activation profile of each synergy was cross-correlated with either the one from synergy A (finger flexor synergy; Figure 9C, D, G, and H) or synergy B (extensor synergy; Figure 9A, B, E, and F) before TT. As previously demonstrated for the FDS and EDC muscles, the temporal activation profiles of muscle Synergies A and B (flexor and extensor) displayed a distinct pattern. After cross-correlation with their own control data, both the flexor and extensor synergies showed coefficients of 1 prior to surgery. These became negative for 66 days post-surgery before reverting close to their original values (Figure 9B and C). However, after cross-correlation with control data of the antagonistic muscle synergy (i.e. extensor for flexor synergy and vice versa, Figure 9A and D), the pattern was reversed. Coefficients started with negative values prior to TT and shifted to positive coefficients near 1 shortly after surgery. In Monkey A, coefficients returned to negative values around day 66 post-TT. In Monkey B, this reversal occurred earlier, around day 48. In short, these results mirror our findings between the transferred muscles (Figure 6G and J). Furthermore, to determine if low correlations were driven by simple timing mismatches, we analyzed the optimal time lag yielding the maximum cross-correlation (Figure 9—figure supplement 1). A positive lag indicates the post-surgery activity is delayed relative to baseline, while a negative lag indicates it is advanced. This analysis revealed significant fluctuations in timing (switching between delays and advances) during the early and mid-adaptation phases, particularly around the ‘switch-back’ period, before stabilizing closer to zero lag in the late phase. For Synergies C (main contributing muscles: ECR, BRD, DEL) and D (main contributing muscles: ECU), the changes in cross-correlation coefficients over time were markedly different. For Synergy C, coefficients begin to steadily increase when cross-correlated with control data of the extensor synergy (Synergy B, Figure 10E) and decrease with the flexor synergy (Synergy A, Figure 10G). This suggests an increasing contribution to finger extension. In contrast, Synergy D does not exhibit any specific pattern following TT.

**Figure 9—figure supplement 1.**
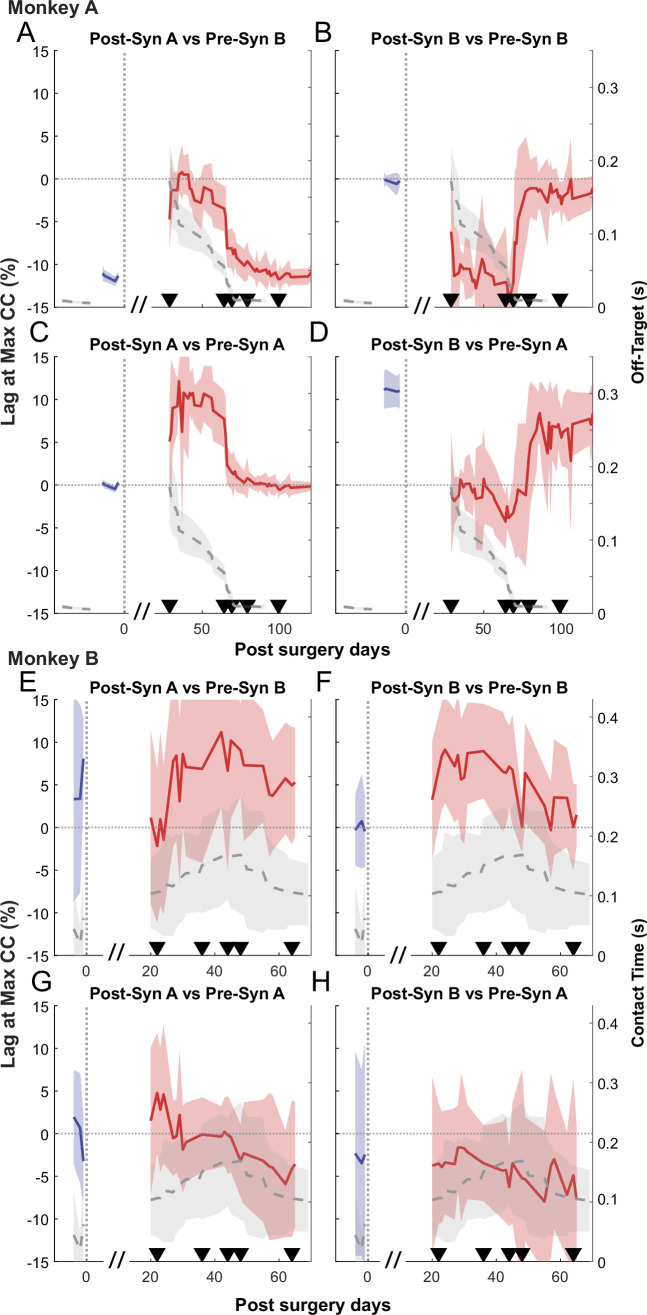
Evolution of time lag at peak cross-correlation between primary synergies. Quantification of the optimal time lag yielding the maximum cross-correlation between the primary flexor (Synergy A) and extensor (Synergy B) synergy activation profiles. (**A–D**) Monkey A; (**E–H**) Monkey B. Plots show the lag between the recorded synergy activation and the pre-tendon transfer (TT) baseline (specific comparisons indicated in titles). Left y-axis: time lag. Blue traces: pre-surgery data; red traces: post-surgery data. Right y-axis (gray dashed traces): behavioral error metrics (off-target reaching duration for Monkey A; contact time for Monkey B). Positive values indicate the synergy activation is delayed relative to the baseline; negative values indicate it is advanced. Note the temporal correspondence between high behavioral cost (peaks in gray traces) and large fluctuations in synergy timing during the early adaptation phase. Shaded envelopes represent standard deviations.

In Monkey B, this cross-correlation analysis revealed a more varied, or differential, pattern of adaptation across the four synergies (Figures 9 and 10). The primary extensor, Synergy B, mirrored the results in Monkey A, showing a clear swap-and-revert pattern (Figure 9F and H). In contrast, Synergy A did not show a clear reversal; its correlation coefficients gradually converged toward zero, likely due to the absence of the FDS EMG signal in the analysis (Figure 9E and G). The secondary synergies, Synergies C and D, showed no discernible trend (Figure 10E–H).

In summary, both monkeys exhibited a distinct two-phase adaptation following TT. We define the ‘early phase’ as the period from initial post-surgical recovery up to the reversal of the swapped activation patterns (approximately days 20/29 to ~65 post-TT, see Figure 9), characterized by the transferred muscles/synergies adopting antagonistic temporal profiles. We also identify a ‘mid-adaptation’ phase covering the transitional period around the ‘switch-back’ event, characterized by high variability. The ‘late phase’ encompasses the period following this switch-back (after ~day 66 post-TT), where original activation timings were largely restored. While this ‘reversion’ toward original activation timings in the late phase marked the abandonment of the initial maladaptive strategy, behavioral recovery involved more than just restoring original patterns. Concurrent with this neural shift in the primary synergies, a distinct compensatory strategy began to emerge, utilizing secondary synergies and biomechanical coupling to achieve functional hand opening, as detailed below.

### The early adaptation phase is characterized by a maladaptive neural and behavioral profile

The early adaptation phase was defined by a distinct neural strategy that correlated with significant behavioral deficits. First**,** the period dominated by off-target reaching movements and prolonged grip times (Figure 5B and E) was precisely when the swapped activation of Synergies A and B was most prominent (Figure 9; see behavioral overlay). This temporal link provides strong evidence that this initial ‘swap’ strategy was, in fact, maladaptive, as the flawed neural control directly underpinned the impairments in movement efficiency and precision.

**Figure 11.**
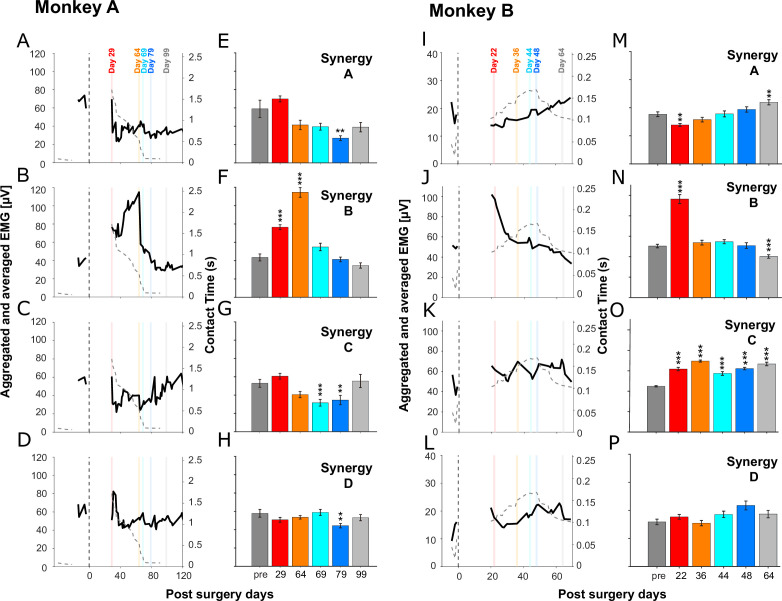
Aggregated and averaged electromyography (aaEMG). aaEMG activities for the main contributing muscles of each synergy for Monkey A (**A–H**) and Monkey B (**I–P**). (**A–D, I–L**) Time course of aaEMG activity (summed within ±15% task range) plotted over post-surgery days. Black dashed lines on the right y-axis indicate behavioral error metrics. (**E–H, M–P**) Bar plots showing the mean (± SEM) aaEMG for the pre-tendon transfer (TT) period (‘pre’) and the five selected landmark days. Vertical colored bars on the time-series plots indicate the corresponding landmark days. Asterisks indicate significant difference from the pre-TT control period (*p<0.01, **p<0.001, ***p<0.0001; two-sample t-test with Bonferroni correction).

Second, we found that the net activity of muscles representing certain muscle synergies (aggregated average EMG [aaEMG]) showed distinct, synergy-specific changes over time (Figure 11). A key feature was the evolution of the activity of finger extensor synergy (Synergy B), which appeared to undergo a neuromuscular ‘arms race’, a rapid escalation of antagonistic co-activation, in both animals, albeit on different timescales (Figure 11B and J). In Monkey A, which struggled for a longer period to regain behavioral proficiency according to our subjective evaluation, our recordings now captured this process unfolding: we observed a steady and significant increase in Synergy B’s aaEMG post-TT until day 64 (p<0.0001) before the strategy changed (Figure 11B and F). In contrast, Monkey B, which adapted its behavior more rapidly according to our subjective evaluation, showed a different profile; its Synergy B activity peaked with a significant surge early in the recording period (day 22: p<0.0001) and subsequently declined toward and below baseline (day 64: p<0.001) (Figure 11J and N). This suggests the initial, rapid escalation of the ‘arms race’ in this animal had already occurred and peaked prior to our first postoperative recording session. Therefore, the apparent discrepancy in the evolution of Synergy B is likely not a fundamental difference in strategy, but rather a reflection of the different observational windows and overall adaptation rates of the two animals.

For the finger flexor synergy (Synergy A), Monkey A showed a general decrease in activity (p=0.0004 at day 79; Figure 11A and E), while Monkey B showed a consistent and significant increase throughout the experiment (day 22: p=0.0004; day 64: p=0.0008). This was expected, as Monkey B’s Synergy A relied on FDP and PL to compensate for the loss of FDS function (Figure 11I and M). Synergy C exhibited a general increase in activity during the post-TT phase in both monkeys (Figure 11G and O), while Synergy D showed no significant differences in Monkey B and only minor changes in Monkey A (Figure 11H and P). All statistical comparisons were made against the pre-TT control period using a two-sample t-test with Bonferroni correction (α=0.01).

Taken together, these distinct patterns of aggregated EMG activity, especially the escalating co-activation within the conflicted Synergy B, a known marker of inefficient muscle recruitment (Thoroughman and Shadmehr, 1999; Osu et al., 2002), further illustrate that this early adaptive phase, which coincided with poor behavioral performance (Figure 5), was characterized by an unstable and inefficient neural control strategy.

### Distinct neural implementations of a compensatory tenodesis strategy

**Figure 12.**
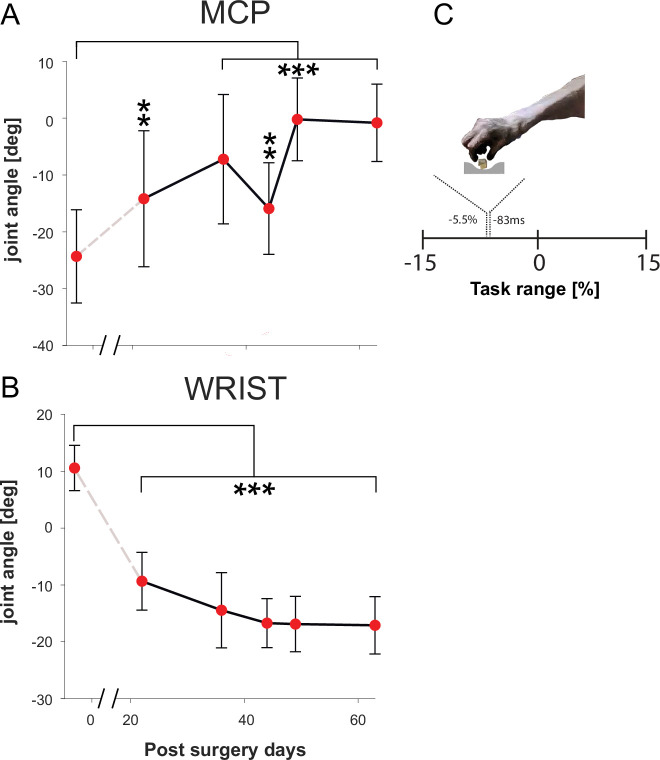
Kinematic analysis of joint angles (Monkey B). Changes in joint angles (mean of 20 trials ± SD) for each landmark day. (**A**) Metacarpophalangeal (MCP) joint angle. (**B**) Wrist joint angle. Asterisks indicate significant difference from pre-tendon transfer (TT) baseline (**p<0.001, ***p<0.0001; ANOVA). (**C**) Schematic indicating the timing of the kinematic snapshot relative to the task timeline (dotted line; 83 ms before food touch), capturing the hand configuration during the pre-shaping phase.

**Figure 13.**
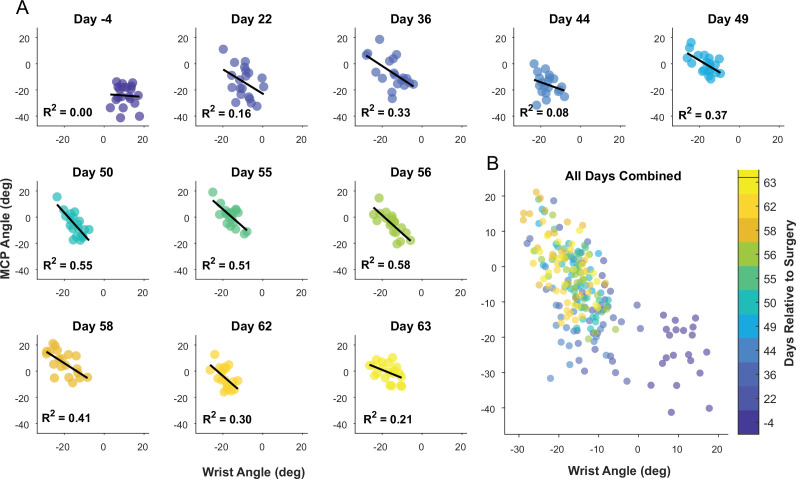
Kinematic analysis reveals gradual refinement of the compensatory tenodesis strategy over time in Monkey B. (**A**) Each subplot shows the trial-by-trial relationship between wrist angle (x-axis) and metacarpophalangeal (MCP) angle (y-axis) for a single recording day (n=20 trials per day). Points are color-coded based on the day relative to surgery (color bar). Pre-tendon transfer (TT) (day –4), no correlation exists (R^2^=0.00). Post-surgery, a negative correlation emerges and strengthens over time, peaking around day 56 (R^2^=0.58), indicating the learned exploitation of the tenodesis effect where wrist flexion predicts finger extension. The tightening of the scatter plots and increase in R^2^ over weeks provide direct evidence for a gradual motor skill learning process. (**B**) All data points combined.

The final phase of adaptation was characterized by the development of a compensatory tenodesis strategy. This effect describes the passive coupling of finger and wrist movements due to the routing of tendons across multiple joints (Zajac, 1992): because the finger extensor tendons pass over the back of the wrist, actively flexing the wrist passively tightens these tendons, which in turn pulls the fingers into extension (Valero-Cuevas and Hentz, 2002; Cash and Jones, 2011). For Monkey B, we found direct kinematic evidence for the acquisition of this strategy. Post-surgery, the monkey learned to significantly increase finger extension at the metacarpophalangeal (MCP) joint (Figure 12A) by concurrently flexing the wrist (Figure 12B; p<0.0001, ANOVA; α=0.01). This coordinated movement pattern is characteristic of an active tenodesis effect. Furthermore, kinematic analysis revealed that this was a gradually learned skill rather than an immediate mechanical consequence. The trial-by-trial coupling between wrist angle and MCP angle (Figure 13) was initially absent (R^2^=0.00) but strengthened over weeks, peaking in the mid-adaptation phase (R^2^=0.58). This period coincided with the stabilization of grasp aperture (Figure 5F) and the resolution of the maladaptive neural patterns (Figure 9). This kinematic strategy was supported by a precise neural implementation. Unlike the scaling strategy observed in Monkey A (see below), Monkey B did not rely on a massive increase in total muscle activity; the aggregated activation of its primary flexor synergy (Synergy A) showed consistent but moderate increases (Figure 11I and M). Instead, the strategy was one of temporal refinement. The activation profile of Synergy A shifted to increase specifically during the pre-contact phase (Figure 7G), providing the necessary wrist flexion for the tenodesis grasp. This was achieved by a precise sequential activation of muscles within the synergy, with the wrist flexor component (PL) peaking just before contact and the finger flexor component (FDP) peaking just after (Figure 6—figure supplement 2). For Monkey A, which successfully restored its primary extensor synergy, the tenodesis grasp likely served as a similar compensatory driver. While we could not perform a kinematic analysis for this animal due to low-resolution video images, strong evidence for this strategy is provided by the neural data. We observed a clear temporal shift in the activation of its dedicated wrist flexor synergy (Synergy C). The peak of this synergy’s activation moved from occurring just after object contact to just before it (Figure 8C), a re-timing well-suited to enable a tenodesis grasp. This increasing contribution is further supported by cross-correlation analysis, which shows its activation pattern became progressively more similar to that of the pre-TT extensor synergy over time (Figure 10A; p<0.05, two-sample t-test).

**Figure 12—figure supplement 1.**
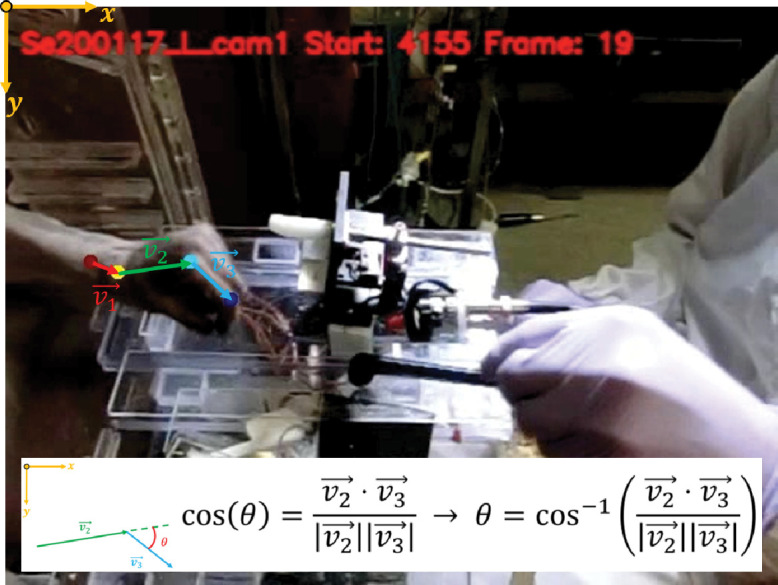
Joint angle measurement. This figure shows an illustration of joint angle measurement using a still picture of the monkey while performing the task. See Methods section for further details.

## Discussion

The CNS’ response to profound musculoskeletal change is a fundamental problem in motor control. This study sought to determine whether the primate CNS adapts to such change by flexibly modulating stable muscle synergies or by developing more fractionated, independent muscle control. We found that the CNS initially defaulted to a modular strategy, repurposing entire synergies by swapping their activation timings. This simple solution, however, proved to be maladaptive, creating a mechanical conflict that impaired motor function under TT surgery used in this study. This early maladaptive phase was ultimately resolved through the gradual development of compensatory movements, leading to a ‘good enough’ functional recovery. This multi-stage process, operating on different timescales, highlights the intricate balance between modularity and flexibility in neural adaptation.

### Neural learning, not physical healing, as the rate-limiting factor

A crucial consideration in interpreting our long-term results is the potential confound of the tendon’s physical healing process. However, the surgical technique employed, a multi-weave Pulvertaft transfer, is designed to provide immediate mechanical strength comparable to that of a native tendon (Graham et al., 2023). While fibrous integration matures over approximately 6 weeks, the repair was biomechanically sound from the beginning. This allows us to conclude that the prolonged, multi-month recovery period, characterized by a complex two-phase neural reorganization, was not limited by the tendon’s force-bearing capacity but rather reflects the significant challenge of a purely neural learning process.

### The CNS defaults to a modular strategy, leading to a maladaptive conflict

Our primary finding provides a direct answer to the question posed in the introduction: the CNS adapted to the TT not by developing fine-grained, fractionated control, but by implementing a modular strategy by repurposing entire co-activation modules (Overduin et al., 2008; Berger et al., 2013; Bizzi and Cheung, 2013; Takei et al., 2017). The initial and most immediate neural change was a wholesale swap of the temporal activation patterns of the primary flexor and extensor synergies (Synergies A and B). The fact that the spatial structure of all four synergies remained remarkably stable throughout the months-long experiment (Figure 7I, Figure 7—figure supplement 2), in the face of drastically altered biomechanics, strongly supports the hypothesis that these synergies represent stable, neurally constrained building blocks (d’Avella et al., 2003; Bizzi and Cheung, 2013; Takei et al., 2017). While our baseline analysis (Figure 7I) confirmed that natural variability is negligible (>0.99 similarity), the specific, transient structural deviations observed post-surgery (e.g. in the compensatory wrist flexor Synergy C) suggest that the CNS retains a limited capacity to fine-tune the internal structure of these modules when driven by strong functional demands. Consequently, the distinct adaptive patterns observed in individual non-transferred muscles (e.g. FCR, PL in Monkey A) likely reflect this task-specific tuning of secondary synergies, rather than a breakdown of modular control.

Our result indicated that this modular approach is likely the default strategy. One potential reason is that modulating pre-existing modules may be computationally simpler and metabolically less costly than developing entirely new, fractionated control patterns (Flash and Sejnowski, 2001; Bizzi and Cheung, 2013). The latter would require extensive synaptic plasticity, potentially involving cortical remapping to selectively uncouple previously co-activated muscles (Kitago and Krakauer, 2013). Alternatively, or perhaps complementarily, the preservation of synergies may reflect inherent constraints on neural plasticity, suggesting that the underlying neural circuits encoding these modules are relatively immutable, even when faced with significant changes in peripheral mechanics (Makin and Krakauer, 2023). Regardless of the underlying reason, be it computational efficiency, constraints on plasticity, or both, the CNS appears to prioritize modulating the activation of existing modules when dealing with acute alterations to the musculoskeletal system.

However, this adherence to established synergy structures created a fundamental conflict. Activating the original extensor synergy (Synergy B) after the transfer now inevitably co-activated the surgically transferred EDC, which functioned mechanically as a flexor, alongside non-transferred muscles like ED23 and ECU, which remained anatomical extensors. This internal mechanical antagonism appears to be the root cause of the early maladaptive phase. This interpretation is supported by two key lines of evidence from our results. First, the period of severe behavioral impairment, characterized by off-target reaching and inefficient grasping (Figure 5B and E), occurred precisely when this flawed ‘swap’ strategy was active. Second, the aggregated EMG activity revealed a sustained and significant increase in the total activation of the conflicted Synergy B in Monkey A (Figure 11B and F), which we interpret as an energetically costly effort to overcome the internal mechanical antagonism. This scenario can be viewed through the lens of optimal control and cost-benefit analysis, where a cost function is minimized (Wolpert, 1997). The initial ‘swap’ strategy, while computationally cheap to select, incurred an unacceptably high operational cost in terms of both poor task performance (high error) and what was likely excessive energy expenditure. We hypothesize that this profoundly unfavorable cost-benefit ratio likely served as the critical error signal that drove the CNS to abandon this initial strategy.

### Resolution through slower compensatory adaptations

The CNS did not persist in the failed swap-based strategy. The high metabolic and computational cost of activating a mechanically conflicted synergy likely triggered the second rapid adaptation: the ‘switch-back’ of synergy activation timings toward their original patterns (Figures 7 and 9). This rapid reversion, occurring over just a few days, is characteristic of an error-based learning mechanism, a form of adaptation that is profoundly impaired by cerebellar damage across a range of tasks, including adaptation to force fields (Smith and Shadmehr, 2005), prismatic shifts (Martin et al., 1996), and split-belt treadmills (Morton and Bastian, 2006). It is plausible that the CNS operates with an implicit threshold for an acceptable cost/performance ratio; once the persistent task failure and high muscular co-contraction of the swap strategy exceeded this threshold, a swift recalibration was initiated. This reversion, despite the remaining mechanical antagonism from the transferred tendons, represents a ‘good enough’ solution where functional success is prioritized over perfect efficiency (Mussa-Ivaldi and Bizzi, 1995; Ranganathan et al., 2013; Gijsberts et al., 2014). Our quantitative analysis supports this interpretation: while the CNS successfully restored the temporal structure of the motor commands (high cosine similarity), it did not perfectly replicate the pre-surgery state. As shown in Figure 7—figure supplement 4, significant differences in activation amplitude persisted in specific phases of the movement, and permutation tests confirmed the profiles remained statistically distinct. This suggests the system settled into a stable, functional attractor that was sufficiently close to the original manifold to execute the task, without expending the computational or metabolic cost required for a perfect restoration. The rapid timescale of this change is highly consistent with the cerebellum’s proposed role as a forward model, predicting the sensory consequences of motor commands and driving rapid learning in response to sensory prediction errors (Popa et al., 2016). Our lag analysis (Figure 6—figure supplement 2) confirmed that the timing of motor commands fluctuated significantly before stabilizing.

Crucially, this switch-back to a less conflicted state, presumably representing the involvement of the error-based learning process, was likely enabled only by the concurrent development of slower, compensatory strategies that provided an alternative means to achieve the task goal. The primary compensation was a learned use of the tenodesis effect. Over weeks, the monkeys gradually increased the activation of the wrist flexor synergy (Synergy C) during hand opening. This wrist flexion biomechanically generates passive finger extension (Zajac, 1992; Cash and Jones, 2011), providing a viable new method for hand pre-shaping. This learned, compensatory behavior, confirmed by kinematic analysis (Figure 12), ultimately allowed the CNS to abandon the maladaptive synergy swap. This gradual, exploratory process is distinct from the following rapid adaptation and represents a form of motor skill acquisition, a process which may be associated with plasticity in cortical structures like the motor cortex and basal ganglia (Kitago and Krakauer, 2013).

### A multi-timescale model reconciles smooth behavioral recovery and abrupt neural reorganization

The apparent paradox between the smooth, gradual recovery of motor function and the abrupt, switch-like reorganization of the underlying neural control strategy suggests that adaptation is not a single process. Instead, we propose it results from the interaction of at least two distinct adaptive systems operating in parallel on different timescales, a concept aligned with established two-state models of motor learning (Smith et al., 2006a; Trewartha et al., 2014).

We map the gradual acquisition of the compensatory tenodesis strategy to the slow process of this model. This iterative component is responsible for building the robust, stable motor skills necessary for long-term recovery. In contrast, we propose that the fast process drives the initial ‘swap' strategy and its subsequent abandonment. This system appears to be sensitive to immediate performance errors and metabolic costs, triggering the rapid ‘switch-back’ to the original synergy timings (recalibration) once the initial strategy proves inefficient.

**Figure 14.**
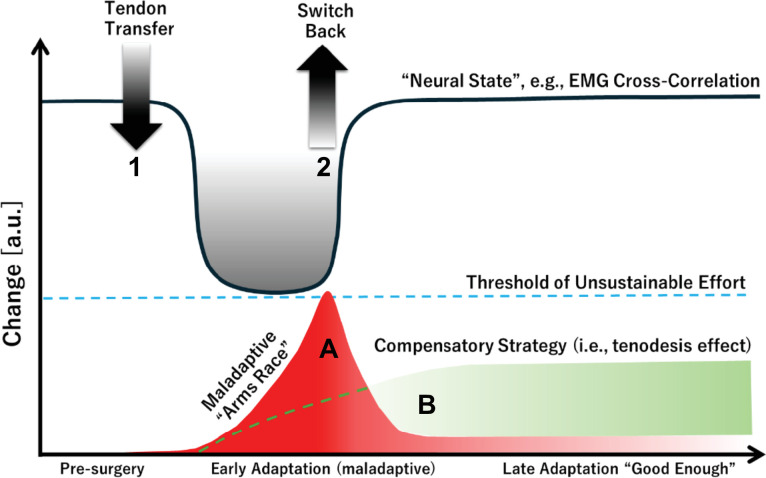
A proposed model of multi-timescale adaptation following tendon transfer. This schematic illustrates the hypothesized interaction between fast and slow adaptive processes driving recovery. The initial tendon transfer triggers a rapid but maladaptive ‘swap’ of motor commands (fast adaptation 1), leading to a maladaptive state. During this phase, two slower processes are hypothesized to occur in parallel: a costly ‘arms race’ within the conflicted synergy (slow process A, red curve) and the gradual development of a functional compensatory strategy (slow process B, green curve). When the ‘arms race’ reaches a threshold of unsustainable cost (dashed blue line), a second fast adaptation (‘switch-back’, 2) is triggered. This allows for the abandonment of the flawed strategy and the adoption of a stable, ‘good enough’ solution, which is now supported by the newly learned compensatory strategy. The gray line represents the observed neural data (e.g. cross-correlation coefficients of electromyography and temporal activation profiles of muscle synergies), which reflects this two-phase process.

The critical insight from our data lies in the interaction between these timescales. We propose that the slow system effectively ‘gates’ the fast system: the abrupt neural reorganization (the switch-back) is likely not an independent event, but a transition enabled only when the slow learning (tenodesis) reaches a ‘good enough’ threshold to support function. This hierarchical interaction resolves the tension between the smooth behavioral curve and the sharp neural transition, ensuring a stable long-term motor plan (Figure 14).

### Broader implications and limitations

#### Mechanisms of neural adaptation

Our findings of a multi-timescale adaptation process involving stable muscle synergies resonate with, yet also extend, previous work on motor learning. Early studies in primates demonstrated a capacity for eventual positive functional readaptation after nerve crossing, moving beyond the initial maladaptive reversals also seen in our monkeys (Sperry, 1947). However, EMG studies in human patients suggest this adaptation may be incomplete, as original ‘old’ muscle activation patterns often persist alongside newly learned ones (Illert et al., 1986). This aligns with our observation of synergy timing modulation rather than structural reorganization, a finding strongly supported by ‘virtual surgery’ studies, indicating the CNS preferentially adapts by recombining existing motor modules (Berger et al., 2013).

#### Biomechanical constraints and sensory feedback

The maladaptive conflict (‘arms race’) observed during the early phase may be exacerbated by peripheral biomechanical factors. Rodent models show that epimuscular myofascial force transmission can cause transferred muscles to generate unexpected antagonistic forces depending on joint angle (Maas and Huijing, 2012). Such biomechanical conflicts likely contributed to the cost and ultimate abandonment of the initial swap strategy. Furthermore, the initial, rapid synergy swap may be linked to a ‘sensory surprise’ from altered proprioceptive feedback, driving a fast but flawed response (Kitazawa et al., 1995; Martin et al., 1996; Edwards et al., 2012; Petitet et al., 2018). This multi-level network reorganization presents a more complex challenge than the gain modulation within single or specific CNS pathways seen in studies of spinal reflex conditioning (Thompson and Wolpaw, 2014). A comprehensive model must ultimately connect the global synergy reorganization with these local, interacting feedback loops, considering factors such as the roles of spinal reflex pathways (Smith et al., 2006b) and fusimotor drive (Vallbo et al., 1979; Hospod et al., 2007).

#### Task-dependent differences

The observation that both monkeys, engaged in both a highly controlled learned task and a more naturalistic grasping task, showed similar adaptive patterns strengthens the generalizability of our findings (Berniker et al., 2013). However, the difference in adaptation rates likely reflects the distinct constraints of these tasks. Monkey A’s precision task required exact force vector production, potentially prolonging the neuromuscular conflict. In contrast, Monkey B’s naturalistic task allowed for more flexible kinematic solutions, such as the tenodesis grasp, to be discovered and employed more rapidly. This suggests that while the core neural mechanism (swap-and-revert) is conserved, the timescale of its resolution is sensitive to task constraints.

#### Motor memory and unlearning

Our paradigm raises critical questions about the distinction between unlearning a maladaptive strategy and relearning a functional one (Kitago et al., 2013). The disappearance of the initial ‘swap’ strategy could be due to passive decay, active inhibition (extinction), or interference from a new, stronger memory (Villalta et al., 2015). The literature increasingly suggests that unlearning is an active process, and that the new, successful compensatory strategy likely supplants the maladaptive one through interference. Our results lend weight to this view, suggesting functional recovery is a process of competitive memory formation.

#### Future directions

A valuable future direction would be to combine the empirical findings of this study with theoretical biomechanical modeling. A subject-specific musculoskeletal model could provide a more precise mapping of how the TT altered the biomechanical plant. Integrating these empirical and theoretical approaches represents a critical next step in understanding the complex interplay between biomechanics and neural adaptation (Nakajima et al., 2023).

### Implications for sensorimotor rehabilitation

These findings may hold implications for sensorimotor rehabilitation. The observation of a distinct maladaptive phase followed by compensation and recovery suggests a staged approach could be beneficial (Hatem et al., 2016; Kakavas et al., 2025). An early phase of therapy might focus on minimizing sensory prediction errors caused by the altered proprioceptive feedback, and reducing maladaptive responses through techniques aimed at integrating the altered body schema (e.g. using visual feedback or task-oriented practice) (Nudo et al., 1996; Takeuchi and Izumi, 2012; Zeiler and Krakauer, 2013; Foell et al., 2014; Levin et al., 2015; De Nunzio et al., 2018; Sattin et al., 2023; van Vliet et al., 2023). This stage would guide the fast, error-driven learning system. A later phase could then employ task-specific training to promote effective compensatory strategies, facilitating the abandonment of maladaptive patterns and the consolidation of a stable motor strategy (Murata et al., 2008; Maier et al., 2019). This second stage engages the slow, skill-acquisition system by creating a rich, problem-solving environment. Finally, a third stage would focus on intensive, high-repetition practice to automate the new functional motor plan, making it robust for real-world use through dosage and generalization (Winterbottom and Nilsen, 2024). This neurobiologically informed staging – guiding fast adaptation, then facilitating slow skill learning, and finally driving consolidation – may offer a more logical and effective path to functional recovery.

## Methods

**Key resources table:** 

Reagent type (species) or resource	Designation	Source or reference	Identifiers	Additional information
Strain (*Macaca fuscata*)	Macaque subjects	National BioResource Project (NBRP)	N/A	Two purpose-bred male subjects (Monkey A: 7.8 kg; Monkey B: 9.9 kg)
Other	AlphaLab SnR system	Alpha Omega Engineering	https://www.alphaomega-eng.com/	Multi-channel data acquisition system for multi-unit EMG signal recording
Other	SONIMAGE MX1	Konica Minolta, Inc	https://www.konicaminolta.com/jp-ja/index.html	High-resolution ultrasound scanning system for real-time muscle and tendon tracking
Other	DS8R	Digitimer	RRID:SCR_024845	Constant current stimulator used for percutaneous muscle activation
Software, algorithm	MATLAB	MathWorks	R2024a; RRID:SCR_001622	Core computing platform for signal filtering, rectification, cross-correlation, and NMF synergy factorization
Software, algorithm	DeepLabCut	Mathis et al., 2018	RRID:SCR_021391	Deep learning framework used for markerless 3D tracking of finger and wrist joint coordinates
Software, algorithm	Kinovea	Kinovea (https://www.kinovea.org/)	N/A	Video analysis software utilized for precision behavioral event time detection

### Animals

Data were collected using two male macaque monkeys (*M. fuscata*; Monkey A: 7.8 kg and Monkey B: 9.9 kg, both 7 years of age) purpose-bred at the National BioResource Project (NBRP). They were kept in custom-made primate cages, allowing for potential pair housing. Both monkeys were trained to perform a simple grasping task. During experimental recordings, the monkeys were seated in a primate chair without further restraint. Their head movement remained unrestricted at all times.

### Procedures

All procedures were designed to minimize discomfort and pain and approved by the Institutional Animal Care and Use Committees at the National Center of Neurology and Psychiatry (NCNP), Tokyo, Japan (ethical approval ID: 2018-009, 2015-006). Details of the surgical operations, experimental setup, and procedures for EMG recordings have been previously described (Takei and Seki, 2010).

### EMG implant surgery

Both animals were familiarized with the experimental setup and trained to perform the behavioral task prior to surgery. After an initial training period, EMG electrodes were chronically implanted subcutaneously into several muscles of the left forearm, including the FDS, FDP, and EDC (Figures 1, 3C, and 4C). Individual muscles were localized and confirmed using electrical microstimulation before implanting EMG electrodes chronically by two methods. For muscles involved in the crossed TT procedure, muscle fascia was cut, and EMG wires with looped ends were placed on top of the muscle belly. These were then secured by reclosing the fascia with absorbable suture threaded through the loop (AS-361, stainless-steel Cooner wire, Conner Wire Co., Chatsworth, CA, USA). All other muscles were directly implanted into the muscle belly using a 22-gauge hypodermic needle. Each wire was threaded into the needle tip, folded back along the shaft, and inserted into the muscle before carefully retracting the needle. Electrode separation for bipolar recordings was approximately 5–10 mm. Note that some EMG recording sites were lost over time. Surgical procedures were carried out under deep general anesthesia (sevoflurane 1.5–2.5% in 2:1 O_2_/N_2_O) and with full aseptic precautions. Heart rate, blood pressure, body temperature, and blood oxygen saturation were monitored throughout surgery. Analgesics and antibiotics were administered intramuscularly for at least 1 week postoperatively.

### TT surgery

After a minimum 4-week recovery period from EMG implant surgery, TT surgery was performed. The FDS and EDC tendons were cut as distally as possible (immediately below and above the wrist for FDS and EDC, respectively) to avoid damaging the Golgi tendon organ located near the junction between the muscle fibers and tendon (Schoultz and Swett, 1972; Jami, 1992). Tendons were then guided either through the gap between the radius and ulnar bone (Monkey A) or around the wrist and then reattached to the tendon of the antagonist muscle using a tendon graft harvested from the plantaris tendon of the lower limb (Monkey B; EDC → FDS: direct connection; and FDS → EDC: tendon grafting). The tendon coaptation was performed using a Pulvertaft weave technique (weaves>2) with high-strength nonabsorbable suture material to ensure immediate and robust mechanical stability post-surgery (Graham et al., 2023). This was only necessary in Monkey B since the tendons used for the cross-transfer were too short. This surgical procedure aimed to reverse the primary mechanical actions of the manipulated muscles, making the FDS tendon an effective finger extensor and the EDC tendon an effective finger flexor. The success and nature of this mechanical rearrangement were verified postoperatively via direct muscle stimulation (see Methods subsection ‘TT confirmation’ and Figure 2). The general organization and function of the major forearm muscles involved in finger flexion and extension in *M. fuscata* are broadly comparable to those in humans (Vanhoof et al., 2021; Yan et al., 2022).

Surgical procedures were carried out under deep general anesthesia (sevoflurane 1.5–2.5% in 2:1 O_2_/N_2_O) and with full aseptic precautions. Heart rate, blood pressure, body temperature, and blood oxygen saturation were monitored throughout surgery. Analgesics and antibiotics were administered intramuscularly for at least 1 week postoperatively. Both monkeys wore a plaster cast post-surgery, but its effectiveness was limited (lasting approximately 1 week).

### TT confirmation

To verify the success and long-term stability of TT surgery, two procedures (Figure 2) were implemented. During the first procedure, the monkey was sedated and its arm was secured to a metal frame (Figure 2A). Electrical stimulation was then applied to either the FDS or EDC muscle (50 mA, DS8R, Digitimer, Welwyn Garden City, UK), while ultrasound scans of tendon movements were concurrently captured (SONIMAGE MX1, Konica Minolta, Inc, Tokyo, Japan; Nordez et al., 2009; Dieterich et al., 2017).

Figure 2B shows a sonogram of the FDS muscle and its intramuscular tendons. The left side displays a static image of the FDS muscle at a specific moment, with white arrows indicating the FDS tendon used for measurement. The right side shows staggered images (top white box) of the FDS tendon displacement triggered by muscle stimulation. The lower right inset illustrates the measurement area. The displacement wave area represents the intensity of muscle contraction and was calculated by measuring the average duration (a, in s) and amplitude (b, in cm) of three successive waves. The area (a*b/2) and regression line for FDS (red) and EDC (blue) (R^2^>0.5 for FDS) for days 0, 7, and 105 post-TT are shown (Figure 2C). The results suggest that the muscle contractions induced by direct electrical stimulation remained nearly constant.

For the second procedure, high-speed videos (37U Series Color Industrial Camera, The Imaging Source, Charlotte, NC, USA) of the monkey’s finger movements were recorded while electrically stimulating the FDS or EDC. Markers were placed on the nails of the index, middle, and ring fingers (Figure 2A) to measure finger displacement in xyz-dimensions using DeepLabCut software (Mathis et al., 2018; Nath et al., 2019). The sum of Euclidean distances of each marker from the origin of the three-dimensional (3D) coordinate system was computed as a scalar quantity. Post-surgery, movement along the z-axis reversed, indicating a shift from finger flexion to extension due to TT (Figure 2D; blue: pre-TT on surgery day, dark brown: post-TT on surgery day, light brown: 1-week post-TT, and red: 3-week post-TT). The scalar quantities of each finger did not significantly change during muscle stimulation on days 0, 7, and 105 days post-TT (Figure 2E), suggesting there was no postoperative tendon rupture or slackening.

### Data recordings

EMG signals were recorded using the AlphaLab SnR system (Alpha Omega Engineering, Hamerkava St. 6, Ziporit Industrial Zone, P.O. Box 810, Nof HaGalil [Nazareth Illit] 1789062, Israel), displayed online, and then stored on a hard drive for later off-line analysis using MATLAB (MathWorks, Natick, MA, USA). Data were recorded at 11 kHz and processed for analysis as follows: (1) down-sampled to 5 kHz; (2) 50 Hz high-pass filtered (sixth-order Butterworth filter); (3) rectified; (4) 20 Hz low-pass filtered (sixth-order Butterworth filter); and (5) down-sampled to 100 Hz. Behavioral task events were recorded as transistor-transistor logic (TTL) signals using the AlphaLab SnR system. Monkey behavior was further recorded by two cameras (Sanyo VPC-WH1, 60 fps [Sanyo, Osaka, Japan]; and Casio EX-100F, 240 fps [Casio, Tokyo, Japan] for Monkeys A and B, respectively) from two different angles (top and side view). The images were later used to detect additional behavioral events (contact time with the object and pull onset for Monkey A; and food touch and food lift onset for Monkey B), and detection of possible changes in the animal’s general movement pattern. Kinovea software was used for this video analysis (free and open-source software; https://www.kinovea.org/).

### Behavioral task

Before and after EMG surgery, the monkeys were trained on a simple grasping task that involved a small object attached to a rod (Figure 3A for Monkey A and Figure 4A for Monkey B). For Monkey A, object 1 was a small rod placed between two side walls encouraging the monkey to grasp the rod with a precision grip using the tips of the index finger and thumb (controlled grasp). The force used to compress the spring while pulling was low and the distance moved short. Object 2 had to be grasped in the same way. However, the force to compress the spring was higher and the distance moved longer (object 1: 300 cN, 4 mm; and object 2: 800 cN, 30 mm). The monkey was asked to grasp and hold the objects for approximately 300–500 ms (Figure 3A–D). After completing the trial, the monkey was rewarded with a piece of fruit that had to be taken from the experimenter’s palm. The task sequence for Monkey A ranged from object 1 hold onset to object 2 hold offset (Figure 3C and D; and ‘Data analysis’ below). For Monkey B, object 1 was a small rod that was grasped and pulled (300 cN, 4 mm) using a lateral prehension grip (like using a door key), and resembling a power grip rather than a natural grasp. However, instead of grasping object 2, Monkey B picked up a piece of fruit from an allocated location. In doing so, the monkey had to cross the path of a photo cell which was detected by the recording system (Figure 4A–D). The task sequence to be analyzed for Monkey B ranged from object 1 hold onset to food touch, which was indicated by an LED (Figure 4C and D; and ‘Data analysis’ below).

For Monkey A, the task sequence was as follows (Figure 3B and Video 1). From a starting position in front of the monkey, it had to lift its arm and move toward object 1, grasp, pull, and then hold the object for 500 ms. Immediately after releasing object 1, Monkey A proceeded to move its hand toward object 2, which was located to the right of object 1. Again, the monkey had to grasp, pull, and then hold the object for another 500 ms. Each hold period was accompanied by an audio signal, which stopped once the hold duration was sufficient. After completing the task, which was signaled by another acoustic signal, the monkey was rewarded with a piece of fruit presented by the experimenter and taken by the monkey.

For Monkey B, the first part of the sequence was identical. However, after releasing object 1, Monkey B was required to pick up a piece of fruit from an allocated location (Figure 4A and B and Video 5). In doing so, the monkey passed a photocell in front of the food well. This event was detected by the recording system and a video camera (a red LED triggered by activation of the photocell). For the analysis, only object 1 and the food grasp were considered in Monkeys A and B, respectively.

### Data analysis

#### EMG analysis

EMG data were normalized to the average time for each monkey to complete a trial (object 1 hold onset → object 2 hold offset/LED offset = 100%). Data were cropped and aligned according to the time stamps (Obj 1 hold offset ± 15%; and LED onset [food touch]±15% for Monkeys A and B, respectively). Recorded EMGs from the pre- and post-tendon surgery period were averaged for each recording and compared over experimental sessions. For Monkey A, FDS and EDC were analyzed both pre- and post-TT. For Monkey B, FDS and EDC were analyzed pre-TT, but only EDC was analyzed post-TT due to signal deterioration.

Both monkeys required time to recover from surgery. Post-TT recordings were resumed once the monkeys were able to perform the task independently (i.e. without assistance from the experimenter) and met specific trial count criteria (starting 29 and 20 days post-surgery for Monkeys A and B, respectively). Postoperative recording sessions were conducted frequently throughout the recovery period, though not at fixed daily intervals, reflecting the practical constraints of long-term behavioral experiments (e.g. animal cooperation, experimental scheduling) and the aim to capture data during key phases of adaptation. Within any given session included in the analysis, behavioral (video) and EMG data were collected concurrently. To be included in the behavioral analyses (e.g. grip formation times, off-target reaching, kinematics), a session required a minimum of 20 successful trials. For the more demanding muscle synergy analyses, a higher threshold of at least 100 successful trials was required to ensure robust factorization. This difference in criteria may have resulted in slightly different sets of recording days being represented across behavioral vs. synergy-related figures. After tendon surgery, EMG signals for FDS, FCU, and FCR deteriorated in Monkey B. Therefore, there is no experimental data for these muscles in this monkey, and they were excluded from the muscle synergy analysis.

#### Synergy analysis

The EMG envelopes obtained from pre-processing raw EMG data were divided by the mean value to normalize activity. Muscle synergies were then extracted for each session using NMF (d’Avella et al., 2003). NMF decomposes the EMG data matrix M, as a product of two matrices C and W:

$$\displaystyle  \rm M = CW^{T} + re = \sum c_i w_i^{T} + re$$

where the vector w_i_ and column of matrix W are muscle synergies; the vector c_i_ and column of matrix C are their temporal coefficients; and re represents the residuals. Multiplicative update rules were used for decomposition (Lee and Seung, 1999). Updating matrices for decomposition from 20 different random initial matrices was started, and factorization results with least residuals among the 20 results were used. To improve robustness of the muscle synergies, one set of muscle synergies was extracted from EMG data of multiple trials of each day. A k-fold cross-validation was used, with k=4, i.e., EMG dataset of multiple trials during the day was split into four datasets, in which three datasets were for training and one dataset was for testing. Synergy matrix W was calculated from three training datasets using NMF, and then a coefficient matrix C was calculated by decomposing the EMG matrix M of the test dataset with a fixed W matrix of the training dataset. This operation was repeated by changing the test dataset for each four datasets. Finally, the extracted four C matrices and four W matrices were averaged to obtain daily synergies.

The number of synergies was determined as the number where VAF exceeded a threshold. This threshold was set as 0.8. To clarify the effect of TT, the number of synergies for each monkey was determined, assuming that trial-by-trial variation in synergies within the same monkey was small. MATLAB ‘nnmf’ function was used for NMF.

To determine whether the weights of muscle synergies changed after TT, the following procedure was used. First, the average of each pre-TT spatial synergy was calculated and used as control data to calculate the cosine distance for all post-TT spatial synergies. This generated four distance relationships for each pre-TT synergy. If the change in spatial synergy before and after TT was small, then only one of the four cosine distances (e.g. synergy A before TT and synergy A after TT) should be significantly smaller. Two-way ANOVA was conducted using the type of synergy and session as factors for the cosine distance. The significance of synergy pairs was identified using the Bonferroni post hoc test.

#### Quantitative comparison of synergy profiles

To quantify the degree of ‘reversion’ in the late adaptation phase, we compared the synergy activation profiles from the pre-surgery period with those from the final recording day using three distinct metrics. First, cosine similarity was calculated to assess the similarity in the shape of the temporal profiles independent of amplitude. Second, a permutation test (n=10,000 iterations) was performed to test whether the specific trajectory of the post-surgery profile was statistically distinguishable from the pre-surgery distribution. Third, to identify specific phases of the task where activation differences persisted, we conducted a point-by-point Wilcoxon rank-sum test at each time point of the normalized task cycle, applying a Bonferroni correction for multiple comparisons.

#### Cross-correlation analysis

Cross-correlation analysis was performed to examine the similarity between pre- and post-surgery EMG signals, as well as temporal activation profiles of extracted muscle synergies. For the EMG signal analysis presented in Figure 6, the cross-correlation coefficient at zero-time lag was calculated. For the synergy analysis presented in the main figures (Figures 9 and 10), the cross-correlation coefficient at zero-time lag was calculated using MATLAB (MathWorks, corrcoef function) to quantify similarity without temporal shifts. These zero-lag coefficients were plotted over experimental sessions. In a supplementary analysis, we also computed the cross-correlation allowing for variable time lags using MATLAB’s xcorr function (normalized using the ‘coeff’ option). From this, we extracted the specific time lag that yielded the maximum cross-correlation coefficient (optimal lag) for each session for both the muscle synergies (Figure 9—figure supplement 1) and the individual EMG signals (Figure 6—figure supplement 1). In this analysis, a positive lag indicates that the post-surgery activation profile is delayed (occurs later) relative to the pre-surgery baseline, while a negative lag indicates it is advanced (occurs earlier).

Based on cross-correlation analysis, five ‘landmark days’ were identified for further analysis (Figure 6P). These days represent distinct stages in the recovery process and excluded pre-TT control data. The first landmark day was chosen from one of the initial recording sessions after TT surgery (days 29 and 22 in Monkeys A and B, respectively) when the cross-correlation coefficients had changed significantly compared with control data. The second day was then chosen from a time period just before the switch-back, when the cross-correlation coefficients had started to return to their original values (days 64 and 36). Another day was then picked from the period when the coefficients were still changing significantly (days 69 and 44) and before starting to saturate. At this point, the next day was defined (days 79 and 48). Finally, the final landmark day was chosen from one of the last recording sessions when the behavior had fully recovered (days 99 and day 64 for Monkeys A and B, respectively).

#### Behavioral analysis

To examine behavioral recovery, the duration, onset, and offset of object and food grasps were analyzed. Event times extracted from the video analysis were used for alignment of EMG data and subsequent cross-correlation and synergy analyses, along with recorded event TTL signals. For each experimental session, the first 20 trials of each video recording were analyzed. In Monkey A, the following events were detected and in-between times stored in ms: touch onset, pull onset, and hold onset (resulting in grasp duration and pull time) for object 1 and object 2. In Monkey B, the following events were detected and in-between times stored: touch onset, pull onset, and hold onset (resulting in grasp duration and pull time) for object 1; and for the food grasp component of the task, LED onset, food touch onset, food lift onset, LED offset, and movement end (contact of food with mouth).

Contact times (touch onset → pull onset) for Monkey A were plotted in ms (mean ± SD) over number of days from tendon surgery for object 1. For Monkey B, contact times with food (food touch → food lift onset) were plotted in ms (mean ± SD).

ANOVA was performed to compare control data recorded before and after tendon surgery. In total, 5 and 3 control sessions vs. 35 and 22 experimental sessions were used for analyses in Monkeys A and B, respectively.

To quantify and compare the baseline behavioral variability of these metrics, we analyzed all pre-TT trials for each animal’s respective tasks. We calculated the CV, defined as the standard deviation divided by the mean, for each metric (grip formation time, pull time, and grasp aperture). To formally test the difference in variance for the grip formation task (Figure 5A and D), we used the non-parametric Ansari-Bradley test. To quantify the observed off-target reaching behavior, video footage was analyzed and the means of 10 consecutive trials calculated for each session as follows. For Monkey A, time spent within a specific spatial window covering the area behind and between the two objects was measured; the monkey passed through this space almost exclusively while exhibiting the impairment (see Figure 3E, crossing of the yellow dotted line). In Monkey B, the time spent in contact with the rear plate of the object was measured (see Figure 4E).

In addition to the primary behavioral metrics, we performed a more detailed video analysis of movement kinematics and performance. For Monkey A, we quantified the ‘pull time’ for each trial, defined as the duration from the moment the monkey started pulling the object to the moment hold onset. For Monkey B, we quantified the ‘grasp aperture’. This was defined as the distance between the tips of the index finger and thumb, measured just before the monkey made contact with the food pellet.

#### Joint kinematics analysis

To determine whether compensatory movements occurred, steady-state changes in joint angles were examined before and after recovery. Using DeepLabCut (Mathis et al., 2018; Nath et al., 2019), key points on the fingers and wrists were tracked from experimental videos. From the obtained key points, the vector v_2_ connecting the wrist and fingers and vector v_2_ connecting the arm and wrist were estimated (Figure 12—figure supplement 1). The joint angle θ was calculated as follows:

$$\displaystyle \rm \cos(\theta)=\frac{\underline{v}_2 \cdot \underline{v}_3}{|\underline{v}_2|\,|\underline{v}_3|} \rightarrow \theta=\cos^{-1}\!\left(\frac{\underline{v}_2 \cdot \underline{v}_3}{|\underline{v}_2|\,|\underline{v}_3|}\right)$$

To quantify the learning and refinement of the compensatory tenodesis strategy over time, we analyzed the trial-by-trial relationship between wrist and MCP joint angles for Monkey B. For each recorded day, we performed a linear regression between the wrist angle (predictor) and the MCP angle (outcome) across all successful trials (mean of 20 trials ± SD for each landmark day, taken 83 ms before food touch). The strength of the coupling was quantified using the coefficient of determination (R²). These daily scatter plots and their corresponding R² values were visualized to track the evolution of the kinematic coupling from the pre-TT baseline through the post-surgery adaptation period.

## Data Availability

All data and custom MATLAB code used to generate the figures in this study are available at the GitHub repository: https://github.com/animalmodel/Philipp_eLife_2025 (copy archived at Philipp and Kosugi, 2026). For long-term preservation, the code and dataset (including large EMG and Synergy matrices) have been archived at Zenodo (DOI: https://doi.org/10.5281/zenodo.18030926). The following dataset was generated: PhilippR KosugiAK 2026animalmodel/Philipp_eLife_2025: Code and data completionZenodo10.5281/zenodo.18030926

## Additional files

Supplementary file 1. Muscle abbreviations, functions, and synergy groupings. Table A: Muscle abbreviations, functions, and synergy groupings. This table provides a reference for all muscles recorded in the study. Columns show the muscle abbreviation, full name, primary functional group, and the main synergy (A, B, C, or D) to which each muscle was assigned for Monkey A and Monkey B. (**place table here)** *Bolded muscles (EDC and FDS) were the targets of the tendon transfer surgery. (¹): EMG signal for this muscle was lost post-surgery in Monkey B and was excluded from the synergy analysis. (N/A): Muscle not recorded or included in the analysis for that monkey*. Table B: Statistical comparison of pre-surgery vs. final post-surgery synergy profiles. (**place table here**) (*Note: Global amplitude refers to a comparison of the distribution of mean activation values across the entire task cycle. n.s.=not significant*).

MDAR checklist
