# Supplementary material for: Multi-timescale neural adaptation underlying long-term musculoskeletal reorganization
Source: eLife. 2026 Jun 19;14:RP108684. doi: 10.7554/eLife.108684 (PMC13282115; doi:10.7554/eLife.108684)
Supplement: Supplementary file 1. — Table A: Muscle abbreviations, functions, and synergy groupings. This table provides a reference for all muscles recorded in the study. Columns show the muscle abbreviation, full name, primary functional group, and the main synergy (A, B, C, or D) to which each muscle was assigned for Monkey A and Monkey B. (place table here) Bolded muscles (EDC and FDS) were the targets of the tendon transfer surgery. (¹): EMG signal for this muscle was lost post-surgery in Monkey B and was excluded from the synergy analysis. (N/A): Muscle not recorded or included in the analysis for that monkey. Table B: Statistical comparison of pre-surgery vs. final post-surgery synergy profiles. (place table here) (Note: Global amplitude refers to a comparison of the distribution of mean activation values across the entire task cycle. n.s.=not significant). [file elife-108684-supp1.docx]

**Supplementary File 1**

**Table A: Muscle Abbreviations, Functions, and Synergy Groupings**

This table provides a reference for all muscles recorded in the study. Columns show the muscle abbreviation, full name, primary functional group, and the main synergy (A, B, C, or D) to which each muscle was assigned for Monkey A and Monkey B.

|  | Full Muscle Name | Primary Function Group | Synergy (Monkey A) | Synergy (Monkey B) |
| --- | --- | --- | --- | --- |
| FCU | Flexor Carpi Ulnaris | Wrist Flexor/Ulnar Deviator | A | N/A¹ |
| **FDS** | **Flexor Digitorum Superficialis** | **Finger Flexor** | **A** | **N/A¹** |
| ECU | Extensor Carpi Ulnaris | Wrist Extensor/Ulnar Deviator | B | D |
| **EDC** | **Extensor Digitorum Communis** | **Finger Extensor** | **B** | **B** |
| ED2,3 | Extensor Digitorum-2,3 | Finger Extensor | B | B |
| FCR | Flexor Carpi Radialis | Wrist Flexor/Radial Deviator | C | N/A¹ |
| PL | Palmaris Longus | Wrist Flexor | C | A |
| BRD | Brachioradialis | Elbow Flexor/Wrist Radial Deviator | D | C |
| ECR | Extensor Carpi Radialis | Wrist Extensor/Radial Deviator | D | C |
| FDP | Flexor Digitorum Profundus | Finger Flexor | D | A |
| DEL | Deltoid | Shoulder Abductor/Flexor | N/A | C |
| ED4,5 | Extensor Digitorum-4,5 | Finger Extensor | N/A | B |

***Bolded muscles (EDC and FDS) were the targets of the tendon transfer surgery.***

***(¹)****: EMG signal for this muscle was lost post-surgery in Monkey B and was excluded from the synergy analysis.* ***(N/A)****: Muscle not recorded or included in the analysis for that monkey.*

**Table B:**

Statistical comparison of Pre-Surgery vs. Final Post-Surgery Synergy Profiles

| Monkey | Synergy | Cosine Similarity (Shape) | Wilcoxon P-value (Global Amplitude) | Permutation Test P-value (Trajectory) |
| --- | --- | --- | --- | --- |
| **Monkey A** | **A** | 0.9243 | 0.3451 (n.s.) | < 0.0001* |
|  | **B** | 0.9483 | **0.0019*** | < 0.0001* |
|  | C | 0.9163 | 0.3808 (n.s.) | < 0.0001* |
|  | D | 0.9433 | 0.3541 (n.s.) | < 0.0001* |
| **Monkey B** | **A** | 0.9043 | **< 0.0001*** | < 0.0001* |
|  | **B** | 0.9726 | **< 0.0001*** | < 0.0001* |
|  | C | 0.915 | **< 0.0001*** | < 0.0001* |
|  | D | 0.9017 | **< 0.0001*** | < 0.0001* |

*(Note: "****Global Amplitude****" refers to a comparison of the distribution of mean activation values across the entire task cycle. "****n.s.****" = not significant).*
