# Supplementary material for: Multi-timescale neural adaptation underlying long-term musculoskeletal reorganization
Source: eLife. 2026 Jun 19;14:RP108684. doi: 10.7554/eLife.108684 (PMC13282115; doi:10.7554/eLife.108684)
Supplement: Peer review history

# Editor report: eLife Assessment

**DOI:** 10.7554/eLife.108684.3.sa0

**Authors:** Juan Alvaro Gallego

**Affiliations:** Champalimaud Foundation Portugal

**Keywords:** Convincing, Important

This **important** study investigates how the nervous system adapts to changes in the mechanics of the body, which are altered through a tendon transfer surgery affecting finger extensor and flexor muscles. By measuring task performance, joint kinematics, and muscle activity for several weeks post surgery, the authors provide **convincing** evidence that monkeys undergo a two-phase adaptation process. First, they adopt a maladaptive strategy to overcome the functional challenges imposed by the surgery, and then revert to a strategy that uses the same patterns of muscle coactivation observed pre-tendon transfer.

# Referee report: Reviewer #1 (Public review)

**DOI:** 10.7554/eLife.108684.3.sa1

Summary:

Many studies have investigated adaptation to altered sensorimotor mappings or to an altered mechanical environment. This paper asks a different but also important question in motor control and neurorehabilitation: how does the brain adapt to changes in the controlled plant? The authors addressed this question by performing a tendon transfer surgery in two monkeys during which the swapped tendons flexing and extending the digits. They then monitored changes in task performance, muscle activation and kinematics post-recovery over several months, to assess changes in putative neural strategies.

Strengths:

(1) The authors performed complicated tendon transfer experiments to address their question of how the nervous system adapts to changes in the organisation of the neuromusculoskeletal system, and present very interesting data characterising neural (and in one monkey, also behavioural) changes post tendon transfer over several months.

(2) The fact that the authors had to employ to two slightly different tasks -one more artificial, the other more naturalistic- in the two monkeys and yet found qualitatively similar changes across them makes the findings more compelling. After all these are very challenging experiments!

(3) The paper is well written, the analyses are sound, and the authors interpret the data appropriately, acknowledging the key limitations.

Weaknesses:

None of note.

# Referee report: Reviewer #3 (Public review)

**DOI:** 10.7554/eLife.108684.3.sa2

Summary:

In this study, Philipp et al. investigate how a monkey learns to compensate for a large, chronic biomechanical perturbation--a tendon transfer surgery, swapping the actions of two muscles that flex and extend the fingers. After performing the surgery and confirming that the muscle actions are swapped, the authors follow the monkeys' performance on grasping tasks over several months. There are several main findings:

- There is an initial stage of learning (around 60 days), where monkeys simply swap the activation timing of their flexors and extensors during the grasp task to compensate for the two swapped muscles.

- This is (seemingly paradoxically) followed by a stage where muscle activation timing returns almost to what it was pre-surgery, suggesting that monkeys suddenly swap to a new strategy that is better than the simple swap.

- Muscle synergies seem remarkably stable through the entire learning course, indicating that monkeys do not fractionate their muscle control to swap the activations of only the two transferred muscles.

- Muscle synergy activation shows a similar learning course, where the flexion synergy and extension synergy activations are temporarily swapped in the first learning stage and then revert to pre-surgery timing in the second learning stage.

- The second phase of learning seems to arise from making new, compensatory movements (supported by other muscle synergies) that get around the problem of swapped tendons.

Strengths:

This study is quite remarkable in scope, studying two monkeys over a period of months after a difficult tendon-transfer surgery. As the authors point out, this kind of perturbation is an excellent testbed for the kind of long-term learning that one might observe in a patient after stroke or injury, and provides unique benefits over more temporary perturbations like visuomotor transformations and over studying learning through development. Moreover, while the two-stage learning course makes sense, I found the details to be genuinely surprising--specifically the fact that: (1) muscle synergies continue to be stable for months after the surgery, despite being maladaptive; and (2) muscle activation timing reverts to pre-surgery levels by the end of the learning course. These two facts together initially make it seem like the monkey simply ignores the new biomechanics by the end of the learning course, but the authors do well to explain that this is mainly because the monkeys develop a new kind of movement to circumvent the surgical manipulation.

I found these results fascinating, especially in comparison to some recent work in motor cortex, showing that a monkey may be able to break correlations between the activities of motor cortical neurons, but only after several of coaching and training (Oby et al. PNAS 2019). Even then, it seemed like the monkey was not fully breaking correlations but rather pushing existing correlations harder to get succeed at the virtual task (a brain-computer interface with perturbed control).

Weaknesses:

I found the analysis to be reasonably well considered and relatively thorough. The authors have also suitably addressed my comments on the previous version. One minor weakness that remains (understandably so) is that the two animals in the study performed different tasks, and the results of the secondary synergy analysis seem to be quite different (Figure 10). That said, I don't think this weakness reduces the impact of the study, and though multiple replications of the same results would provide more convincing evidence, I don't think it's necessary to make the points that the authors are making.

# Author comment: Author response

**DOI:** 10.7554/eLife.108684.3.sa3

**Authors:** Roland Philipp, Yuki Hara, Naohito Ohta, Naoki Uchida, Tomomichi Oya, Tetsuro Funato, Kazuhiko Seki

**Affiliations:** National Institute of Neuroscience, Department of Neurophysiology, NCNP Tokyo Japan; National Center of Neurology and Psychiatry, Department of Neurophysiology Tokyo Japan; National Center of Neurology and Psychiatry, Department of Neurophysiology Tokyo Japan; National Center of Neurology and Psychiatry, Department of Neurophysiology Tokyo Japan; Western University London Canada; University of ElectroCommunications Tokyo Japan; National Center of Neurology and Psychiatry, Department of Neurophysiology Tokyo Japan

The following is the authors’ response to the original reviews.

**Public Reviews:**

**Reviewer #1 (Public review):**

(1) I think this is an important paper, but I’m puzzled about a tension in the results. On the one hand, it looks like the behavioural gains post-TT happen rather smoothly over time (Figure 5). On the other hand, muscle synergy activations change abruptly at specific days (around day ~65 for Monkey A and around day ~45 for Monkey B; e.g., Figure 6). How do the authors reconcile this tension? In other words, how do they think that this drastic behavioural transition can arise from what appears to be step-by-step, continuous changes in muscle coordination? Is it “just” subtle changes in movements/posture exploiting the mechanical coupling between wrist and finger movements, combined with subtle changes in synergies, and they just happen to all kick in at the same time? This feels to me to be the core of the paper and should be addressed more directly.

We thank the reviewer for this insightful comment, as it touches upon the central finding of our study. The apparent tension between the smooth behavioral recovery and the abrupt shift in neural strategy is indeed a key feature of the adaptation process. We propose that this reflects the interaction of two distinct, parallel processes operating on different timescales:

A slow, gradual skill-learning process, where the monkeys incrementally developed and refined a compensatory motor strategy (i.e., the tenodesis effect). This slow refinement is responsible for the smooth improvement seen in the behavioral metrics over many weeks.

A fast, switch-like adaptive process, which governs the activation of the primary muscle synergies. The initial ‘swap’ strategy, while simple, was biomechanically conflicting and inefficient. The CNS only abandoned this flawed strategy abruptly once the slow learning process had rendered the new compensatory strategy “good enough” to be a viable alternative.

Therefore, the abrupt neural shift does not cause the behavioral improvement but is rather enabled by the gradual, underlying development of a better motor solution. To address this important point more directly within the manuscript, we added a new subheading to the Discussion section. This section is dedicated to explicitly framing our findings within this multi-timescale learning model, ensuring the link between the gradual behavioral recovery and the abrupt neural shift is clearly articulated.

(2) The muscle synergy analyses, which are an important part of the paper, could be improved. In particular:

(a) When measuring the cross-correlation between the activation of synergies, the authors should include error bars and should also look at the lag between the signals.

We thank the reviewer for these excellent suggestions to improve our analysis.

Error Bars: We agree that showing trial-to-trial variability is important. In our revision, we have added a shaded envelope (representing the SD across trials) to the cross-correlation plots in Figures 6, 9 and 10.

Time Lag: We have performed the cross-correlation analysis allowing for variable time lags and extracted the lag yielding the maximum correlation coefficient (max CC) for each session, in addition to the zero-lag correlation presented in the main figures. As hypothesized, allowing variable lags often resulted in high max CC values throughout the adaptation period, potentially obscuring the clear swap-and-revert pattern visible in the zerolag analysis. This is likely because the primary adaptation involved changes in synergy timing rather than fundamental shape. However, the analysis of the lag itself proved informative. We observed significant fluctuations in the optimal lag during the early and mid-adaptation phases, particularly around the time of the ‘switch-back’, before the lag stabilized closer to zero in the late phase.

We have added a description of this analysis to the Methods section. The results of the lag analysis are now presented in a new Supplementary Figure S6 and S7, and a sentence summarizing this finding has been added to the Results section.

(b) Figure 7C and related figures, the authors state that the activation of muscle synergies reverts to pre-TT patterns toward the end of the experiments. However, there are noticeable differences for both monkeys (at the end of the “task range” for synergy B for monkey A, and around 50% task range for synergy B for monkey B). The authors should measure this, e.g., by quantifying the per-sample correlation between pre-TT and post-TT activation amplitudes. Same for Figures 8I, J, etc.

We thank the reviewer for this detailed and insightful suggestion. We agree that our use of the term ‘reversion’ should be nuanced, as the recovery of the synergy activation patterns is substantial but not perfect.

To formally quantify these remaining differences, we performed a rigorous quantitative comparison between the pre-surgery and final-day post-surgery activation profiles. We calculated the Cosine Similarity to assess the recovery of the temporal shape, and used a Permutation Test (n=10,000) to test for statistical distinctness between the pre- and post-surgery trajectories.

Results: We found that while the temporal shapes were highly similar (Cosine Correlation > 0.90 for all synergies), the Permutation Test confirmed that the profiles remained statistically distinct (p < 0.0001) in both animals.

We have added this quantification to the text (Results). This confirms our nuanced interpretation: while the primary temporal features of the synergies reverted, the recovered motor program represents a novel, ‘good enough’ solution that is robust and functional, rather than a mathematically perfect restoration of the original baseline.

(c) In Figures 9 and 10, the authors show the cross-correlation of the activation coefficients of different synergies; the authors should also look at the correlation between activation profiles because it provides additional information.

We thank the reviewer for this comment and the opportunity to clarify our terminology. We agree that analyzing the correlation between the full activation profiles is the most informative approach. In our manuscript, the terms ‘activation coefficients’ and ‘activation profiles’ both refer to the complete, time-varying activation patterns of the muscle synergies. Therefore, the crosscorrelation analysis presented in Figures 9 and 10 is indeed the correlation between these full activation profiles. To prevent any potential ambiguity for future readers, we have revised the manuscript to use the term ‘activation profiles’ exclusively and consistently when referring to these time-varying synergy activations.

(d) The muscle synergy analysis for Monkey B is hindered by the fact that the authors lost the ability to record from the (very) functionally relevant FDS muscle. I’d repeat the synergy analyses without this muscle to understand to what extent the observed changes with respect to baseline are driven by the lack of this data.

We thank the reviewer for raising this important methodological point. We agree that controlling for changes in the recorded muscle set is crucial for a valid comparison between pre- and post-surgical synergy structures. The reviewer’s concern is based on the premise that the FDS muscle was included in the pre-surgical analysis for Monkey B but absent from the postsurgical analysis.

We would like to clarify that this is not the case. Due to the loss of the FDS signal post-surgery, we made the deliberate decision to exclude the FDS muscle from ALL synergy analyses for Monkey B, including the pre-surgical baseline period. This was done for the precise reason the reviewer identifies: to ensure a direct and unbiased “apples-to-apples” comparison and to avoid introducing the lack of this muscle as a confound. Therefore, the changes in synergy structure that we report for Monkey B can be confidently attributed to genuine physiological adaptation rather than an artifact of a changing input dataset.

(e) Figure 11: The authors talk about a key difference in how Synergy B (the extensor finger) evolved between monkeys post-TT. However, to me this figure feels more like a difference in quantity - the time course than quality, since for both monkeys the aaEMG levels pretty much go back to close to baseline levels - even if there’s a statistically significant difference only for Monkey B. What am I missing?

We thank the reviewer for this insightful question, as it has prompted us to refine our interpretation of this key finding. The reviewer correctly notes that the recovery trajectories of Synergy B appear different, and we agree that our original explanation can be improved.

A more parsimonious interpretation, and one that we believe aligns better with the data, is that both monkeys likely underwent a similar ‘arms race’, but we captured different phases of this process. In Monkey A, our recordings (starting Day 29) captured the escalating phase of this neuromuscular conflict. In contrast, for Monkey B, recordings began on Day 20, by which time this rapid escalation had likely already occurred and peaked. This difference in the timing of the ‘arms race’ is consistent with our behavioral observations; Monkey A struggled for a longer period before performing the task proficiently, suggesting a more protracted overall adaptation process. Thus, the apparent difference in the figures is likely a reflection of the observational window and the individual adaptation rate of each animal, rather than a fundamental qualitative difference in their adaptive strategy. We have revised the text to present this more unified and coherent interpretation.

(f) Lines 408-09 and above: The authors claim that “The development of a compensatory strategy, primarily involving the wrist flexor synergy (Synergy C), appears crucial for enabling the final phase of adaptation”, which feels true intuitively and also based on the analysis in Figure 8, but Figure 11 suggests this is only true for Monkey B. How can these statements be reconciled?

We believe the reviewer may be referring to Monkey A in their comment, as the strong compensatory effect is indeed seen in this animal. The core of this issue, which we have clarified in our revision, is that both monkeys developed a compensatory tenodesis grasp but used different neural strategies to achieve it.

For Monkey A, strong evidence for this strategy is provided by a clear temporal shift in the activation of its dedicated wrist flexor synergy (Synergy C). As we have now clarified in the manuscript, the peak of this synergy’s activation moved from occurring just after object contact to just before it, a re-timing well-suited to enable a tenodesis grasp.

For Monkey B, the strategy was one of subtle re-timing rather than scaling. While the total aggregated activation of its primary flexor synergy (Synergy A) did not significantly increase, its temporal profile shifted. Specifically, activation prior to object contact increased, providing the necessary wrist flexion for its assistive tenodesis grasp, which was kinematically confirmed in Figure 12. This was achieved by reallocating activation from the post-contact phase, resulting in an earlier activation peak for the synergy overall. Crucially, a finer-grained analysis reveals a precise temporal sequence within this synergy’s activation: the wrist flexor component (PL) consistently peaked just before object contact to enable hand opening, while the finger flexor component (FDP) peaked just after contact to secure the grasp.

This timing resolves the apparent biomechanical conflict. It also reveals that while both monkeys converged on the same biomechanical solution (a tenodesis grasp), the observable neural implementation appeared different. However, we must be cautious in directly comparing the computed synergy structures themselves, as the analysis for Monkey B was performed without the FDS muscle. The apparent “multi-functional synergy” in Monkey B is most likely a consequence of this missing data. What is clear and robust, however, is that both monkeys converged on a remarkably similar temporal solution: they both learned to re-time the activation of their key wrist flexor muscles to the pre-grasp phase.

In Monkey A, this was observed in the temporal shift of its dedicated wrist flexor synergy (Synergy C). In Monkey B, this was observed in the temporal shift of the Palmaris Longus (PL) muscle itself (which, in our computed synergies, was grouped into Synergy A). This convergence on an identical temporal adaptation, regardless of the computed modular organization, is the key finding. We have revised the manuscript to articulate this more precise and defensible interpretation.

(3) Experimental design: at least for the monkey who was trained on the “artificial task” (Monkey A), it would have been good if the authors had also tested him on naturalistic grasping, like the second monkey, to see to what extent the neural changes generalise across behaviours or are task-specific. Do the authors have some data that could be used to assess this even if less systematically?

We thank the reviewer for raising this important point regarding the generalizability of our findings across different behaviors. We fully agree that a direct comparison of both tasks in the same animal would have been a valuable experiment. Unfortunately, we do not have systematic data on naturalistic grasping for Monkey A that would allow for such a direct comparison. We therefore view the two tasks as providing complementary evidence. Monkey A’s data shows the adaptation process during a highly stereotyped behavior, while Monkey B’s data demonstrates that a similar two-phase adaptive process occurs during a more naturalistic, unconstrained task. The convergence of these findings strengthens our overall conclusion that this multi-timescale adaptation is a robust principle of motor learning. Nonetheless, the reviewer raises a fascinating question about the task-specific tuning of motor synergies, which remains an excellent direction for future studies.

(4) Monkey B’s behaviour pre-tendon transfer seems more variable than that of Monkey A (e.g., the larger error bars in Figure 5 compared to monkey A, the fluctuating crosscorrelation between FDS pre and EDC post in Figure 6Q). This should be quantified to better ground the results since it also shows more variability post-TT.

We thank the reviewer for this excellent suggestion to formally quantify the presurgery behavioral variability. We have performed the suggested analysis on the "Grip Formation Time" metric (Fig. 5A), which was the comparable metric between the two tasks. Our calculation of the Coefficient of Variation (CV) confirms the reviewer’s observation. Monkey B’s pre-surgery performance was substantially more variable (CV = 81.93%) than Monkey A’s (CV = 46.62%). Furthermore, a non-parametric test for equal variances (Ansari-Bradley test) confirmed that this difference is highly statistically significant (p < 0.0001). We have added a description of this analysis to the Methods and reported this finding in the Results section to provide a clearer context for the baseline differences between the subjects.

(5) Minor: Figure 12 is interesting and supports the idea that monkeys may exploit the biomechanical coupling between wrist and fingers as part of their functional recovery. It would be interesting to measure whether there is a change in such coupling (tenodesis) over time, e.g., by plotting the change in wrist angle vs change in MCP angle as a scatter plot (one dot per trial), and in the same plot show all the days, colour coded by day. Would the relationship remain largely constant or fluctuate slightly early on? I feel this analysis could also help address my point (1) above.

We thank the reviewer for this excellent and insightful suggestion. We have performed the suggested analysis for Monkey B, plotting the trial-by-trial relationship between wrist and MCP angles for all recording days (New Figure 13).

The results clearly show the gradual refinement of the tenodesis coupling. Pre-surgery, there was no correlation (R²=0.00). Immediately post-surgery (Day 22), the relationship was weak and variable (R²=0.16), reflecting an exploratory phase. Over the following weeks, the coupling became progressively stronger and more consistent, with the R² value peaking at 0.58 around Day 56, indicating a robust exploitation of the new strategy. The relationship then stabilized at a moderate level (R² ~0.2-0.3) in the final days. This analysis provides direct kinematic evidence for the slow, gradual skill-learning component of our two-state model. It beautifully complements our response to the reviewer’s first point by visualizing the underlying refinement process that occurred concurrently with the more abrupt neural shifts. We have added this new figure and a description of these results to the manuscript.

**Reviewer #2 (Public review):**

Weaknesses:

The most notable weakness of the study is the incompleteness of the data. [...] As a result, it is difficult to make general conclusions from the study, and it awaits further analysis or the addition of another subject.

We thank the reviewer for this critical and accurate assessment of the study’s limitations. The reviewer is correct that the datasets for the two monkeys are incomplete in different ways and that the tasks were not identical. We fully acknowledge these limitations throughout the manuscript. Rather than viewing these differences as a weakness that prevents generalization, we propose that they offer a unique strength in the form of complementary evidence. We consider the two animals not as a direct replication, but as two distinct case studies that test the same underlying hypothesis under different conditions.

Monkey A, with its high-quality EMG and highly stereotyped task, provides a detailed, quantitative view of the neural adaptation process, allowing us to precisely characterize phenomena like the ‘neuromuscular arms race’.

Monkey B, with its kinematic data and more naturalistic task, provides crucial evidence that the same fundamental principles, a two-phase adaptation and the eventual development of a compensatory strategy, generalize to a less constrained, more behaviorally relevant context. We believe the key finding is the convergence of the results. Despite the differences in individual strategy, task demands, and available data, both animals demonstrated the same core "swapand-revert" adaptive process. We propose that this convergence from heterogeneous sources lends support to the generalizability of our conclusions, suggesting that the multi-timescale adaptation we describe may be a general feature of motor learning following such perturbations. We agree that future studies with more subjects are needed to fully establish this principle. Nonetheless, we feel that the convergent evidence from these two complementary cases provides a valuable foundation for the model we present.

A second weakness is the insufficient analysis of the movements themselves, particularly for Monkey A. [...] Since the authors have video data for both monkeys, it is surprising that it was not used to extract landmarks for kinematic analysis, or at least hand/endpoint trajectory, and how it is adjusted over time. Adding more behavior data and aligning it with the EMG data would be very helpful for characterizing motor recovery and is needed to support conclusions about underlying neural control strategies for functional improvement.

We thank the reviewer for this important suggestion. The reviewer’s comment prompted us to re-examine our behavioral data, and we have now performed additional analyses that we agree provide a much clearer link between the neural changes and functional recovery.

For Monkey A, we have quantified the ‘pull times’ on a day-by-day basis. This analysis reveals a clear, gradual learning curve: pull times were initially long and variable post-surgery but steadily decreased and stabilized over the recovery period. This provides a direct, quantitative measure of motor performance recovery for this animal.

For Monkey B, we have performed a detailed analysis of the ‘grasp aperture’ prior to object contact. This kinematic analysis is particularly revealing, as it shows the development of the compensatory strategy in real-time. The grasp aperture was initially very small post-surgery, reflecting the monkey’s inability to open its hand. It then steadily increased over the next ~40 days as the monkey learned and refined the compensatory tenodesis grasp, before stabilizing at a new, functional baseline.

We believe these new analyses directly address the reviewer’s concern by providing a more detailed picture of motor recovery. The grasp aperture data, in particular, offers a clear kinematic correlate for the slow, skill-learning process that we propose runs in parallel to the more abrupt neural reorganization. We have added these results as a new figure in the main text of our revised manuscript.

Considering specific conclusions, the statement that the monkeys learned to use “tenodesis” over time by increasing activation of a wrist flexor muscle synergy does not seem to be fully supported by the data. [...] Given these issues, it is not clear how to align the EMG and kinematic data and interpret these findings.

We thank the reviewer for this detailed and critical analysis. They raise an excellent point and have correctly observed that the adaptation is not a simple, uniform increase in wrist flexor synergy amplitude. Our interpretation, which we have clarified in the manuscript, is that the monkeys learned a more sophisticated strategy: a precise re-timing of the wrist flexor activation to occur earlier in the movement, specifically to pre-shape the hand for the grasp.

For Monkey A: The reviewer correctly notes that the peak amplitude of Synergy C (the wrist flexor synergy) around the moment of grasp (0% task range) is lower in the final phase compared to baseline. However, the crucial change is temporal: the peak of this synergy’s activation shifts from occurring just after the grasp (~+1%) to occurring just before it (~-2%). This re-timing is perfectly suited to enable finger extension via the tenodesis effect immediately prior to object contact. The subsequent lower amplitude may reflect a more efficient, less forceful movement once this new skill was refined.

For Monkey B: The reviewer is right that this monkey does not have a dedicated wrist flexor synergy and that the overall amplitude of the PL muscle does not increase dramatically. However, a closer look at its activity profile (Fig. S2-AN) reveals a clear and consistent increase in activation specifically in the pre-contact phase (~7% task range). This is the precise neural signature of the assistive tenodesis grasp that is kinematically confirmed in Figure 12. The monkey is not simply scaling up the synergy; it is strategically activating it earlier to prepare for the grasp.

In summary, the key evidence linking the EMG to the tenodesis strategy is in the temporal domain. The learned re-timing of the wrist flexor activation to the pre-grasp phase is the crucial link that aligns the neural and kinematic data. We have revised the manuscript to make this distinction between amplitude scaling and temporal shifting clearer.

A more minor point regarding conclusions: statements about poor task performance and high energy expenditure being the costs that drive exploration for a new strategy are speculative and should be presented as such. Although the monkeys did take longer to complete the tasks after the surgery, they were still able to perform it successfully and in less than a second and no measurements of energy expenditure were taken.

We thank the reviewer for this important point regarding the precision of our language. We agree that statements regarding ‘high energy expenditure’ and the specific drivers for exploring a new strategy are interpretations of the data, not direct measurements, and should be framed as such.

Our speculation about energetic cost is based on the significant increase in muscle co-activation we observed (e.g., Fig. 11), a phenomenon widely understood to be metabolically expensive. Similarly, while the monkeys were still successful, their prolonged movement times and inefficient motor patterns represent a clear performance deficit compared to their highly optimized presurgical baseline, which we propose acted as a driver for further adaptation. In our full revision, we have carefully revised the manuscript to soften these claims. We have used more speculative language, such as “we hypothesize that...”, “the likely cost of...”, or “may have provided the impetus for...” to ensure that our interpretations are clearly distinguished from our direct empirical findings.

A small concern is whether the tendon transfer effect may fail over time, either due to scar tissue formation or tendon tearing, and it would be ideal if the integrity of the intervention were re-assessed at the end of the study.

We thank the reviewer for raising this important point regarding the long-term integrity of the tendon transfer. We agree that a terminal anatomical re-assessment would be an ideal control. While a terminal assessment was not performed as part of this study’s protocol, we were able to monitor the transfer’s integrity throughout the study. We are confident the transfer remained functionally intact for two key reasons:

(1) Physical Monitoring: We periodically used ultrasound imaging to non-invasively visualize the tendon repair, which allowed us to confirm its continued physical integrity.

(2) Functional Evidence: This physical confirmation was corroborated by the functional data. Both animals achieved stable, proficient task performance that was maintained for months. Furthermore, the late-phase neuromuscular control strategies became highly consistent. A significant failure, such as a tendon tear or prohibitive mechanical scarring, would be incompatible with this sustained behavioral and neural stability.

Nevertheless, we agree that a terminal assessment is an excellent methodological suggestion that should be incorporated into the design of future long-term studies of this nature.

**Reviewer #3 (Public review):**

(1) First, I find myself wondering about the physical healing process from the tendon transfer surgery and how it might contribute to the learning. Specifically, how long does it take for the tendons to heal and bear forces? If this itself takes a few months, it would be nice to see some discussion of this.

We thank the reviewer for this insightful question about the potential contribution of the physical healing process to the adaptation timeline. Our surgical protocol was specifically designed to ensure the tendon transfer was biomechanically robust from the outset, minimizing the role of healing as a rate-limiting factor.

We used a Pulvertaft weave technique, which is known to achieve mechanical strength equivalent to that of a native tendon shortly after the procedure (Graham et al., 2023). The repair involved more than two weaves and utilized high-strength suture material to maximize its initial forcebearing capacity. While full fibrous integration around the suture site typically occurs within approximately six weeks, the repair itself was strong enough to bear physiological forces immediately post-surgery. Therefore, the prolonged, complex, two-phase multi-month behavioral recovery and the neural reorganization we observed cannot be attributed to a slow physical healing process. Instead, this supports our conclusion that the observed timeline reflects the challenges and constraints of a purely neural adaptation and skill-learning process. To make this crucial point clear to all readers, we have added these details about the surgical method to the Methods section and included a brief discussion of its implications in the Discussion.

(2) Second, I see that there are some changes in the muscle loadings for each synergy over the days, though they are relatively small. The authors mention that the cosine distances are very small for the conserved synergies compared to distances across synergies, but it would be good to get a sense for how variable this measure is within synergy. For example, what is the cosine similarity for a conserved synergy across different pre-surgery days? This might help inform whether the changes post-surgery are within a normal variation or whether they reflect important changes in how the muscles are being used over time.

We thank the reviewer for this excellent and insightful suggestion. Establishing a baseline for normal day-to-day variability is an important control for our synergy analysis.

We have performed this analysis in full. Specifically, to quantify baseline stability, we calculated the cosine similarity between the spatial synergy weights (W) of each individual recording day and the pre-surgery average. This provides a rigorous measure of day-to-day variability relative to the stable baseline structure. We have added these data to Figure 7 (Panel I), which plots the pre-surgery similarity (blue traces) alongside the post-surgery adaptation (red traces).

We found that baseline stability was remarkably high, with cosine similarity consistently exceeding 0.99 (e.g., Monkey A: 0.99 ± 0.001). This quantification allows the reader to formally assess that the changes observed post-surgery (e.g., drops to ~0.80 or ~0.60 in Monkey B) are well outside the range of normal physiological fluctuation, representing subtle but genuine structural adaptation.

(3) Last, and maybe most difficult (and possibly out of scope for this work): I would have ideally liked to see some theoretical modeling of the biomechanics so I could more easily understand what the tendon transfer did or how specific synergies affect hand kinematics before and after the surgery. Especially given that the synergies remained consistent, such an analysis could be highly instructive for a reader or to suggest future perturbations to further probe the effects of tendon transfer on long-term learning.

We thank the reviewer for this excellent and forward-thinking suggestion. We completely agree that a detailed biomechanical model of the tendon transfer would be a powerful tool for understanding the mechanical consequences of the surgery and for interpreting the function of the recorded muscle synergies. However, creating a subject-specific musculoskeletal model with the fidelity required to accurately simulate synergy-to-kinematic transformations is a highly complex project that we feel is well beyond the scope of the current manuscript. Such an endeavor would constitute a major research project in its own right.

Our study’s primary focus was to provide a detailed, longitudinal characterization of the in-vivo neural adaptation following this perturbation, a dataset that is itself rare and valuable. We aimed to document the physiological learning process as it unfolded over many months. Nonetheless, the reviewer’s point is exceptionally well-taken. Currently, we are constructing a monkey musculoskeletal model and performing tendon transfer on this model to investigate what kind of characteristics in the learning process reproduce the synergy changes observed in the experiments. Although this project is still in progress, to date, we have demonstrated that the robustness of synergies themselves is necessary for changes in muscle activity at the synergy level (Nakajima N, Wang S, Ogihara N, Oya T, Seki K, Funato T, Upper Limb Musculoskeletal Model of Macaque Monkey for Approaching Adaptation Mechanism to Tendon Transfer, Society for Neuroscience 2023, Washington DC, USA, 2023).

The rich dataset we have collected in the present research could serve as an excellent foundation for developing and validating such a model in the future. We believe that combining these two approaches is a critical and exciting next step for the field, and we have highlighted this as a key future direction in our discussion.

**Recommendations for the authors:**

**Reviewing Editor Comments:**

When revising the manuscript for resubmission, please try to improve the visual presentation of the data, which is a point highlighted by all three reviewers during the discussion, including making the presentation of monkey-specific results more consistent across subjects.

We have comprehensively revised the figures to ensure a consistent and clear visual presentation, as requested. Specifically, we standardized the layout across all main and supplementary figures (placing Monkey A consistently in the top rows or left columns and Monkey B in the bottom rows or right columns) and applied unified color schemes throughout the manuscript. Furthermore, we harmonized the presentation of the analytical results, such as the specific cross-correlation pairings in Figures 9 and 10, to ensure that the data for both subjects are presented with identical logic, facilitating direct comparison.

**Reviewer #1 (Recommendations for the authors):**

(1) Please revise the writing; some words are missing (line 90), and some sentences could be clarified slightly, even if the paper is well written (lines 317-320). The paragraph including the idea of tenodesis could also be further clarified, I think.

Thank you for pointing these out. We have corrected the missing word (osteoarthritis) on line 90. We have also revised lines 317-320 to remove ambiguity. Furthermore, the section describing the tenodesis effect (now section "Distinct neural implementations...") has been substantially rewritten for improved clarity, incorporating a more detailed explanation of the biomechanics.

(2) In the Introduction, the authors cite Hunter and Eckstein 2009 and Mercuri and Muntoni 2013 without describing the pathological conditions; this will not be clear for not nonspecialists.

Thank you. We have added brief descriptions ("osteoarthritis, a degenerative joint disease," and "muscular dystrophy, which involves progressive muscle weakness,") directly into the Introduction sentence where these references appear.

(3) Data presentation: I often thought that the data could be presented more clearly:

(a) For example, Figure 3D and 4D should show error bars around the mean to have a sense of the consistency of pre-lesion behaviour. Same for other figures like Figure 6.

We appreciate the reviewer's suggestion to visualize data consistency. (a) Figures 3D, 4D, and 6 (EMG Profiles): For these figures, we opted to display mean traces and peak markers to clearly illustrate the temporal shifts and relationships between muscles. Overlaying multiple standard deviation envelopes in these comparative plots would significantly reduce legibility. However, to fully address the reviewer's request to see the consistency of pre-lesion behavior, we direct attention to Supplementary Figure S1, which presents the complete EMG profiles with full error tubes (Mean ± SD) for every recorded muscle. (b) Quantitative Analysis Figures: We ensured that variability is explicitly visualized in all statistical analyses. The crosscorrelation time-courses in Figures 6 (G-Q), 9, and 10 are plotted with shaded error tubes to show variance. Similarly, the aggregated EMG analysis in Figure 11 utilizes bar plots with explicit error bars to quantify the statistical consistency of the changes.

(b) The autocorrelation analysis in Figure 6 should also include measures of lag if it’s not at zero lag. If it’s the latter, please specify it in the Methods.

We thank the reviewer for this question regarding the cross-correlation analysis presented in Figure 6 (Panels G-J, P-Q). We confirm that this analysis was performed at zero time lag. To clarify this, we have added a sentence to the Methods section (Subsection "Crosscorrelation analysis") explicitly stating that the EMG cross-correlations shown in Figure 6 were calculated at zero lag. We have also added a clarifying note ("at zero time lag") to the description of these panels within the Figure 6 caption.

(c) Seeing EMG patterns similar to those presented in Figures 3D and 4D at different times post-lesion (e.g., as a Supplementary figure) would also give readers a better intuition of the neural changes.

We thank the reviewer for this suggestion to provide more intuitive examples of the neural changes. We realize we did not sufficiently highlight this in the main text, but this complete data is already available in the manuscript. Supplementary Figures S1 and S2 provide a comprehensive overview of the EMG patterns for all recorded muscles in Monkey A and Monkey B, respectively. These figures show the pre-surgery and post-surgery average profiles for all recording sessions as well as the average profiles from five different post-surgery landmark days, covering the entire adaptation period. We have added explicit cross-references to these figures in the main text.

(d) I couldn’t fully understand the analysis in Figure 4E; clarify.

We thank the reviewer for noticing this oversight. The reviewer is correct that Figure 4E was not referenced in the main text. This panel was intended to show the baseline kinematic profiles (MCP and wrist angles) for Monkey B's control session, corresponding to the average EMGs shown in panel 4D. Given that our more comprehensive kinematic analyses are now presented in Figure 12 and the new Figure 13, we believe panel 4E is largely redundant. To improve the clarity and focus of Figure 4, we have removed panel 4E and its description from the revised manuscript.

(e) Some figures showing neural changes (e.g., Figures 6G-J, 6P,Q, Figures 9 and 10, and even Figure 11 for different reasons) would become more understandable if they were accompanied by the behavioural changes (e.g., something like Figure 5A on top of them).

We agree that visualizing the temporal link between neural reorganization and behavioral recovery is essential for interpreting the data. We have implemented this suggestion by overlaying behavioral metrics onto the right y-axes of Figures 6 (G-Q), 9, 10, and 11. However, regarding the specific behavioral metric, we opted to overlay the maladaptive behavior/aberrant reaching metric (from Figure 5B) rather than the grip formation time (Figure 5A). We found that the maladaptive behavior profile provided a clearer and more direct correlate to the neural data, as its peak coincides precisely with the ‘swapped’ synergy phase, thereby effectively illustrating the functional cost of that specific neural state.

(f) Some figure captions could be improved by adding more detail (e.g., for Figure 6).

We agree. We have substantially expanded and improved the captions for Figure 6 and Figure 7 to make them more self-contained and guide the reader more effectively through the key findings presented in the panels. We have also reviewed other captions for clarity.

(g) I’d show the cosine distance between synergies across days as a main figure, e.g., as part of Figure 7, because this is an important result.

We agree that the longitudinal stability of the synergy structures is a crucial result that deserves prominence. We have implemented this suggestion by adding a new panel, Figure 7 (I, K) for primary synergies and Figure 8 (K, L) for secondary synergies, which plots the cosine similarity of the spatial synergy weights across the entire experimental timeline. This figure explicitly visualizes the high stability of the pre-surgery baseline (blue traces, similarity > 0.99) and contrasts it with the dynamic structural tuning observed during the post-surgery adaptation (red traces), providing a clear, day-by-day account of synergy evolution as requested.

(h) In Figure 7C, D and G, H, it’d be interesting to also see in the background the EMG for the transferred muscle that belongs to each synergy, to appreciate their relationship.

We thank the reviewer for this suggestion. To illustrate the close relationship between the primary synergies and their key constituent muscles, while avoiding visual clutter in the complex post-surgery plots, we have modified the pre-surgery panels of Figure 7 (C, D, G, H). In these panels, we have now overlaid the average pre-surgery EMG profile of the primary transferred muscle belonging to that synergy (e.g., FDS for Synergy A, EDC for Synergy B) as a thin, gray, dashed line. This visually confirms the tight correlation between the synergy profile and the muscle’s activity at baseline.

(i) In page 10, the authors report as maladaptive behaviour the duration of the aberrant reaching component from day 29 (monkey A) and day 20 (monkey B). What was happening before those recording dates? Were the monkeys recovering?

Thank you for this question. We have added two sentences to the start of the Results section (“Functional Recovery Follows...”) clarifying that the period between surgery and formal recordings included approximately one week of home cage recovery followed by several weeks of assisted task practice. Formal recordings began once the monkeys could perform the task consistently without assistance.

(j) In the Methods (EMG Analysis), the authors state that they resumed their recordings post-TT “once they (the monkeys) were able to perform the task on their own”. It would be good if the authors made this more precise (e.g., based on success rate or another metric).

We thank the reviewer for this suggestion to increase precision. We have revised the Methods section to include the specific criteria used for resuming post-surgical recordings. Recordings were restarted once the monkeys were able to perform the task independently (i.e., without assistance from the experimenter) and consistently achieved a successful trial count of at least 100 trials within a single experimental session.

(k) Line 266- reads “Alternation of EMG activity in non-transferred muscle suggests one possibility: TT might alter the control strategy of coordinated muscle activity for hand movement by modifying the transferred muscles and their agonists as a cohesive unit”, however, some “muscles showed patterns that were incompatible with a simple swap” (Lines 255-256). Doesn’t this observation suggest that what happens is not a simple change in muscle synergies?

We thank the reviewer for this insightful question regarding the interpretation of muscles with adaptive patterns incompatible with the primary ‘swap-and-revert’. We agree that these observations require careful consideration within the modular framework. Our interpretation is that these muscles do not represent evidence against modular control, but rather reflect the involvement of multiple modules adapting concurrently. Specifically, muscles like FCR and PL, which showed distinct patterns, are primary members of Synergy C (the wrist flexor synergy) in Monkey A. Their adaptive profile is therefore consistent with the task-specific recruitment and retiming of Synergy C as part of the compensatory tenodesis strategy, rather than being a deviation from the swap observed in Synergies A and B. Synergies represent the dominant, shared variance in muscle activity. While they capture the overall strategy, some degree of individual muscle variation or the influence of secondary synergies is expected. We have added a sentence to the Results section to clarify that these diverse patterns likely reflect the differential involvement of muscles in multiple adapting synergies. We believe the overall evidence still strongly supports the modulation of stable synergies as the primary mechanism of adaptation in this paradigm.

(l) You may want to call synergy A and synergy B, synergy F and synergy E to make recall easier? (Same for synergy C and D, which could be F2 and E2).

We thank the reviewer for this helpful suggestion aimed at improving clarity. We considered renaming the synergies based on function (e.g., F/E). However, given the number of figures and the complexity of a global change, and the fact that the functional roles of Synergies C and D differed between animals, we decided to retain the original A/B/C/D labels for consistency. To ensure clarity for the reader, we have carefully checked the manuscript to ensure that we consistently define the primary functional role of each synergy (e.g., "Synergy A, the primary finger flexor synergy") when it is discussed.

(m) Lines 315-317 - “These pattens of changes in synergy 3 and 4, both contributed minimally to the EMG of transferred muscles” -> This statement puts the causality as synergies cause muscles to activate according to certain patterns, which is supported by work by several groups -including the authors- however, they could also reflect biomechanical and task constraints as other have argued; perhaps this tone would be better for the discussion?

We thank the reviewer for this nuanced point regarding the interpretation of synergy contributions. We agree that the causal relationship between computed synergies and muscle activity is complex and can reflect both neural commands and task constraints. To address this, we have revised the sentence in question in the Results section. Instead of stating that the synergies "contributed minimally," we now state that the changes in these synergies "were associated with minimal EMG activity in the transferred muscles." This phrasing is more descriptive of the observation and less implicitly causal, while retaining the key point within the flow of the results. The subsequent sentences, which offer interpretation, are already framed speculatively ("This suggests...", "may have served...").

(n) Line 403 How do the authors conclude from the synergy patterns in Figure 11 that the early post-TT is characterised by “an unstable and inefficient neural control strategy”? To me, this is shown clearly in the behaviour, not in these plots, unless I’m missing something?

We thank the reviewer for this comment, which highlights the need to clearly connect our neural findings to the behavioral outcome. The reviewer is absolutely correct that the behavioral data (Fig. 5) provides the most direct evidence of instability and inefficiency during the early adaptation phase. Our intention was to argue that the neural patterns observed in Figure 11 provide a physiological correlate for this behavioral inefficiency. Specifically, the escalating aggregated EMG activity observed in the conflicted extensor synergy (Synergy B), which we term the ‘arms race’, represents significant muscle co-activation. Such co-activation is widely understood to be energetically costly and reflects a suboptimal control strategy where the CNS is essentially "fighting itself" against the altered mechanics. To make this link clearer, we have revised the concluding sentence of the relevant paragraph in the Discussion ("The early adaptation phase...") to explicitly state that this escalating co-activation is a known marker of inefficient recruitment and that it occurred concurrently with the period of poor behavioral performance shown in Figure 5.

(o) Lines 469-471. The authors suggest that muscle synergies may be preserved post-TT because a modular approach (to motor control) may be computationally easy and metabolically cheap. To me, recent data suggest that the most parsimonious explanation is what they later say: that the nervous system may not be plastic enough to change this (e.g., see Makin and Krakauer, “Against reorganisation” also in eLife).

We thank the reviewer for raising this important theoretical point and for referencing the relevant literature on constraints on cortical reorganization. We agree that the preservation of muscle synergies in the face of such a profound perturbation is a key finding that warrants careful interpretation. In our revised Discussion (section "The CNS Defaults to a Modular Strategy..."), we have now explicitly incorporated the perspective that synergy stability may reflect inherent constraints on neural plasticity, citing Makin and Krakauer (2023), alongside our original hypothesis regarding computational and metabolic efficiency. We present these ideas not as mutually exclusive, but as potentially complementary factors that both contribute to the CNS’s apparent preference for modulating existing modules rather than fundamentally restructuring them.

(p) Lines 501-503. Also on interpretation. Would the metabolic cost indeed be much higher? Couldn’t the observed change in strategy be explained purely based on performance metrics?

This is an important point. We agree that statements regarding high energy expenditure are interpretations, not direct measurements. We have carefully revised the manuscript (Abstract, Results, and Discussion) to soften these claims, using more speculative language (e.g., "likely costly," "what we propose was...") to clearly distinguish our interpretations from direct empirical findings.

(q) Lines 538-. The authors link the initial adaptation phase to the fast process reported in adaptation studies and say that this leads to poor retention. However, it seems from their data that the behaviour is stable across (early) days, so doesn’t this rule out such an interpretation?

We thank the reviewer for this insightful question regarding the interpretation of the early adaptive phase within the two-state model framework. The reviewer correctly notes that the early post-surgical behavior, while maladaptive, appeared relatively stable across days and did not show the rapid decay sometimes associated with the "poor retention" characteristic of the fast system. We agree that this apparent stability requires careful interpretation. In our revised Discussion (section "A Multi-Timescale Model..."), we now propose that the fast system is primarily responsible for the initial, rapid adoption of the ‘swap’ strategy in response to the large error signal. The subsequent persistence of this flawed but stable state for several weeks is likely not due to strong retention by the fast system itself, but rather reflects the time required for the parallel slow system to gradually develop a more effective compensatory strategy (i.e., the tenodesis grasp). Once this alternative strategy became viable, it enabled the abrupt "switchback," which we also attribute to the fast system recalibrating away from the highly costly swap strategy. Therefore, we believe our data is consistent with the involvement of a fast system driving rapid strategic shifts, even if the typical "poor retention" phenotype is masked by the lack of a viable alternative strategy during the early phase.

**Reviewer #2 (Recommendations for the authors):**

(1) The discussion would benefit greatly from a more careful comparison with prior work characterizing the response to experimental or clinical tendon or nerve transfer in different models.

We thank the reviewer for suggesting these important references and for the recommendation to compare our findings more carefully with prior work. This is an excellent point, and we agree it will significantly strengthen the discussion. In our full revision, we have added a new paragraph to the Discussion section dedicated to this comparison. We discuss how our findings relate to classic work showing primate adaptive capacity beyond simple maladaptive responses (Sperry, 1947), EMG evidence for the persistence of original neural patterns alongside new ones in human patients (Illert et al., 1986), the critical role of altered peripheral biomechanics and myofascial force transmission in complicating adaptation (Maas & Huijing, 2012), and how our observation of synergy stability aligns with evidence for modular adaptation strategies (Berger et al., 2013). This comparison helps situate our unique findings of a multi-timescale process and synergy timing modulation within the broader context of motor relearning after musculoskeletal rearrangement.

(2) Line 90 - Which disease or condition is studied in Hunter and Eckstein (2009)?

Thank you. We have clarified this in the Introduction; the reference pertains to osteoarthritis.

(3) Line 280 for clarity in text and as a reminder to the readers, please state which muscles are involved in each synergy grouping.

We have updated the text (Results, 'Adaptation occurs through modulating...') to explicitly list the main contributing muscles for each synergy grouping (e.g., Synergy A: FDS and FCU for Monkey A). This provides the requested clarity regarding the functional identity of each synergy while maintaining readability. For the complete, quantitative muscle weight composition including minor contributors, we referred the reader to Figure 7 and Supplementary Table 1.

(4) Line 180 There are differences in the time course for measurements between the behavioral metrics and EMGs. If not recorded at fixed time intervals, the differences in the time courses for the two monkeys should be explained.

We thank the reviewer for this question regarding the time courses of our measurements. We interpret this comment in two ways, both of which we have addressed in the revised manuscript.

First, if the reviewer is asking about the overall recording schedule, they are correct that sessions were not performed at fixed daily intervals, and the specific days sampled differed between monkeys. This non-uniform sampling was due to the practical constraints of longterm behavioral experiments (e.g., animal cooperation, scheduling, weekends) and the aim to capture data during key phases of adaptation. However, within any given session, behavioral (video) and EMG data were always collected concurrently.

Second, if the reviewer is asking whether the set of days included differs between the behavioral plots (e.g., Fig 5) and the EMG/synergy plots (e.g., Figs 6, 9-11), this is a possibility depending on data quality criteria. Our criterion for including a session in the behavioral analysis was a minimum of 20 successful trials. However, for the more demanding synergy analysis, we required a higher minimum of 100 successful trials to ensure robust factorization. It is possible that a few sessions met the behavioral criterion but not the synergy criterion and were thus excluded from the latter analysis, leading to slight differences in the days presented across figures. To ensure full clarity, we have added text to the Methods section explicitly stating: (A) the rationale for the non-uniform daily sampling schedule, and (B) the specific minimum trial count criteria used for including data in the behavioral versus the synergy analyses, noting if this resulted in different sets of days being analyzed for different figures.

(5) General figure comments - The figures are informative, but they could be better presented, designed, and formatted to explain the important results in the paper. The figures should be able to explain most of the key results without entirely referring to the text to find some of the details. I had a bit of trouble understanding Figure 9 & 10. I would also like to suggest that bringing raw data into some figures (e.g., EMG of different muscle groups), such as showing stability between the synergies, could improve the results and allow the story to flow with more clarity. Likewise, clearly showing the differences between baseline EMG measurements and post-surgery measurements could improve some of the result figures.

We thank the reviewer for these important general comments on data presentation. We agree that the figures are the key to our story and are implementing several revisions based on this and other reviewer feedback to improve their clarity.

General Presentation: We have conducted a thorough review of all figures to improve layout, consistency, and font legibility (addressing R3, 1 and the Reviewing Editor's comments). This includes adjusting the layouts of Figures 3, 4, and 6 for better alignment and clarity.

Figures 9 & 10 (Cross-correlation): The reviewer mentioned having trouble understanding these figures. In our revision, we have substantially rewritten the captions for Figures 9 and 10 to be much more descriptive. We explicitly walk the reader through how to interpret the plots (e.g., "The ‘swap’ is evidenced by the drop in self-correlation... and a concurrent rise in antagonist-correlation...").

Including "Raw Data" (EMG): We thank the reviewer for this suggestion to provide more intuitive examples of the neural changes. We realize we did not sufficiently highlight this in the main text, but this complete data is already available in the manuscript. Supplementary Figures S1 and S2 provide a comprehensive overview of the EMG patterns for all recorded muscles in Monkey A and Monkey B, respectively. These figures show the pre-surgery and post-surgery average profiles for all recording sessions as well as the average profiles from five different post-surgery landmark days, covering the entire adaptation period. These figures directly visualize the swap-and-revert pattern in the transferred muscles and their agonists (e.g., EDC, ED23), as well as the diverse and complex adaptations in other nontransferred muscles (e.g., FCR, PL), as requested. To make this clearer, we have added explicit cross-references to Supplementary Figures S1 and S2 within the main Results section to ensure readers are directed to this detailed data.

Showing Differences (Pre vs. Post): To "clearly show the differences between baseline... and post-surgery measurements," we implemented the point-by-point statistical comparison of pre- vs. final-day synergy profiles (as suggested in R1, 2b). This has resulted in a new Supplementary Figure visually highlighting the precise periods in the task where the final profiles still differ significantly from baseline (Fig. S9).

We believe these additions (new figures and improved captions) will make the results much clearer and more self-explanatory, as the reviewer suggested.

(6) Figure 1 A table with all the acronyms would help with identifying all the muscles and their respective synergies (supplemental), especially when describing the muscles in the result of the discussion section.

This is an excellent suggestion. We have created a comprehensive table (Supplementary Table 1) listing all muscle abbreviations, full names, primary functional groups, and assigned synergies for both monkeys. We have added a reference to this table in the Figure 1 caption and the Methods section.

(7) Figure 2 - is this mainly from Monkey A? If so, it should be stated.

We thank the reviewer for pointing out this omission. We have updated the caption for Figure 2 to clarify that the example data shown (ultrasound, trajectories, and quantitative plots) are from Monkey A.

(8) Figure 3 & Figure 4 seems unbalanced because of the descriptive need to explain Monkey B’s tasks? The figure alignments could be better.

We thank the reviewer for this comment on the visual presentation of Figures 3 and 4. The reviewer’s observation that the figures appeared ‘unbalanced’ was correct. This was a direct consequence of two issues: (1) the different tasks required slightly different schematics (the "descriptive need" the reviewer mentioned), and (2) the original Figure 4 contained an additional kinematic panel (formerly 4E) that was unique to Monkey B, which broke the parallel structure with Figure 3.

To address this and significantly improve the alignment, we have now moved the unique kinematic panel (formerly 4E) to a new Supplementary Figure (Supplementary Figure S8). This change has allowed us to re-arrange the panels in Figures 3 and 4 so that they now follow the exact same order. We have also adjusted the layout to ensure that corresponding panels are of a consistent size. We agree that this creates a much better visual balance and makes the comparison between the two monkeys far more direct and clear, as the reviewer suggested.

(9) Figure 5. It seems like the animals can still perform the task post-surgery, but with high variability. Maybe emphasize the differences in variability between baseline and postsurgery?

We thank the reviewer for this suggestion to emphasize the changes in variability. We have now quantified this using the Coefficient of Variation (CV) for key behavioral metrics across different phases (Pre-surgery, Early, Mid, Late post-surgery). The results confirm the reviewer’s observation of high variability post-surgery, particularly in the early phase. For instance, Monkey A’s grip formation time CV spiked dramatically (Pre: 47% vs Early: 133%), while Monkey B’s remained high (Pre: 82% vs Early: 76%). Interestingly, while Monkey A’s variability returned close to baseline levels in the late phase (Late: 55%), Monkey B’s variability increased further (Late: 97%), suggesting persistent inconsistency despite functional recovery.

We also observed metric-specific changes. Monkey A’s pull time became less variable than baseline later on (Pre: 65% vs Late: 43%), suggesting refinement of that action. Conversely, Monkey B’s grasp aperture remained consistently low throughout (Pre: 26% vs Late: 19%), indicating relatively precise kinematic control was maintained or quickly regained. We have added a summary of these findings to the Results section to provide a more complete picture of how behavioral variability evolved relative to baseline during the adaptation process.

(10) Figure 6 quite a confusing figure. This figure needs to be better presented. The figure legends are hard to see for Monkey A vs Monkey B. At first, I thought Monkey B’s figure legend also represented Monkey A. I would suggest reorganizing the figures for clarity and coherence.

We agree that the original presentation of Figure 6 was dense and potentially confusing. We have completely reorganized the figure to improve clarity and coherence.

(1) Clear Separation: The figure is now structured with a strict separation between Monkey A (Left Panels, A-J) and Monkey B (Right Panels, K-Q), with prominent headers for each subject to prevent ambiguity.

(2) Improved Legends: We have redesigned the legends to be larger and placed them explicitly within their respective subject’s section to ensure it is immediately clear which data they describe.

(3) Visual Consistency: We have standardized the color schemes and axis layouts across this and all other figures to reduce cognitive load and facilitate easier comparison between subjects.

(11) Figure 12 - This figure is incomplete without Monkey A’s results. The videos in the supplemental sections seem clear enough for some kinematic analysis. The story could be more supported with more thorough measurements of the kinematics from both animals to show how they differ over time and by highlighting the two phases. As a minor note, it would be helpful to present the kinematic data together with a schematic of when during the task the data are drawn from, using the % task range scale, since that is the standard throughout the paper.

We thank the reviewer for their suggestions regarding the kinematic analysis. We agree that a parallel kinematic analysis for Monkey A, similar to that in Figure 12, would be ideal. We did attempt this. Unfortunately, while the supplemental videos for Monkey A are sufficient for observing the overall movement trajectory, they are not suitable for the detailed joint angle analysis the reviewer suggests. The videos for Monkey A were recorded at an insufficient frame rate that did not allow to reliably extract the rapid joint angle positions of the wrist and fingers during the grasping movement. This is the reason why this detailed kinematic analysis was limited to Monkey B, for which we had high-speed video recorded at 240 fps, allowing for a robust analysis of these fast movements.

We have, however, expanded our kinematic analysis for Monkey B to show the refinement of the tenodesis strategy over the full time course (New Figure 13), which does help to highlight the different adaptive phases for that animal. We have also clarified in the manuscript (e.g., in the caption for Figure 12) that the lack of Monkey A data for this specific analysis was due to the lowresolution and low-frame-rate video available.

We agree that defining the precise timing of the kinematic snapshot relative to our normalized task range is critical for accurate interpretation. In response, we have added a new panel (Figure 12C) that explicitly maps the kinematic snapshot to our standardized task timeline. This schematic clarifies that the joint angle analysis captures the hand configuration during the pre-shaping phase, specifically at 83 ms prior to object contact (which corresponds to -0.02% of the normalized task range). This ensures the kinematic data can be directly interpreted within the same temporal context as the EMG and synergy results presented throughout the paper.

**Reviewer #3 (Recommendations for the authors):**

First and most major: I found many of the figures much too small and incredibly difficult to read. Possibly the most difficult was Figure 7, where I had to zoom in a great deal to read what muscles corresponded to which bars. I don’t have specific suggestions here other than to make sure that figures are legible.

We thank the reviewer for highlighting this important issue. We have comprehensively revised the figures to ensure they are legible at standard publication sizes. Specific improvements include:

(1) Figure 7: We have significantly increased the font size of the x-axis muscle labels and optimized the bar chart spacing to ensure the muscle identities are readable without excessive zooming.

(2) Global Updates: Across all figures, we have increased font sizes for axis labels and titles, removed unnecessary whitespace to maximize the data-to-ink ratio, and exported all final figures in high-resolution vector formats to ensure clarity.

Second and more minor: I liked the setup of the manuscript, where the authors explained the unique benefits of their experimental methods and the question they were going after (“When confronted with structural changes to the musculoskeletal system, does the CNS adapt by modulating existing synergies, or by shifting toward more fractionated control strategies?”). However, the evolution of the paper made the answer to this question seem very confusing to me as I read it. The results show that monkeys initially modulated existing synergies in phase 1, but then reverted to the original modulation. This, in addition to the way the question was set up initially, made me think the conclusion was going to be that the synergies themselves changed in the second phase, but this paradoxically was not the case--synergies were stable throughout. I was left confused for the back half of the results section, until the discussion on tenodesis and developing compensatory movement strategies. So the answer is that the monkey learns by modulating existing synergies, but using different strategies in different learning phases. I’m not entirely sure how to avoid this confusion, but I wonder if there’s a way to foreshadow this finding earlier on.

We thank the reviewer for this valuable feedback on the manuscript’s narrative structure. We understand how the initial framing (modulation vs. fractionation) followed by the reversion of the initial modulation could lead to confusion before the compensatory strategy is fully introduced. To address this, we have made two key adjustments in the revised manuscript:

(1) In the Introduction, after posing the central question, we have added a sentence to subtly foreshadow that the adaptive process might be complex and multi-phasic, requiring analysis over extended timescales.

(2) In the Results section, at the transition point between describing the reversion of the primary synergy timings and introducing the compensatory tenodesis strategy, we have added a short paragraph to explicitly signal that the reversion was not the complete solution and that a distinct compensatory strategy emerged concurrently.

We believe these changes improve the narrative flow, provide better signposting for the reader, and mitigate the potential for confusion identified by the reviewer, making it clearer that the ultimate solution involved modulating existing synergies but via different strategies across distinct learning phases. We appreciate the reviewer’s help in identifying this area for improvement.
